# The EU pollinator hub controlled vocabulary: An ontology for pollinators

**DOI:** 10.1371/journal.pone.0353382

**Published:** 2026-08-26

**Authors:** Michael Rubinigg, Gregor Sušanj, Gilles San Martin, Jordan Benrezkallah, Awad Hassan, Noa Simon Delso

**Affiliations:** 1 Dr Mag. Michael Rubinigg, Frohnleiten, Austria; 2 BeeLife European Beekeeping Coordination, Bruxelles, Belgium; 3 Združba IP, d.o.o., Maribor, Slovenia; 4 Centre Wallon de Recherches Agronomiques, Gembloux, Belgium; 5 Laboratory of Zoology, Research Institute for Biosciences, University of Mons, Mons, Belgium; 6 Mellivory, Egypt; Ladoke Akintola University of Technology, NIGERIA

## Abstract

The Pollinator Ontology (PolOn), a subset of the EU Pollinator Hub Controlled Vocabulary (EUPH-CV), is an ontology for information and data on pollinators and their interactions with the abiotic and biotic environment, particularly humans. It contains a series of classes, their properties and the relationships between them. A first version of the ontology has been deployed as part of a web-based open-source software application, the EU Pollinator Hub, a tool that has been developed, among others, to promote (1) the standardisation and internationalisation of data related to pollinators, (2) the compliance of this data with FAIR guiding principles for scientific data management and stewardship, and (3) the community-driven development of the ontology in the future.

## Introduction

Pollinators are not only objects of study in biological research, but also play essential ecological, agronomic and economic roles [1,2]. Hence, data related to pollinators stretches across a variety of domains. On the other hand, data on pollinators are often fragmentary or lacking because collected data cannot be efficiently shared or because a lack of standardisation, interoperability, and suitability for machine processing impairs their availability for analysis [3–5].

**Fig 1:**
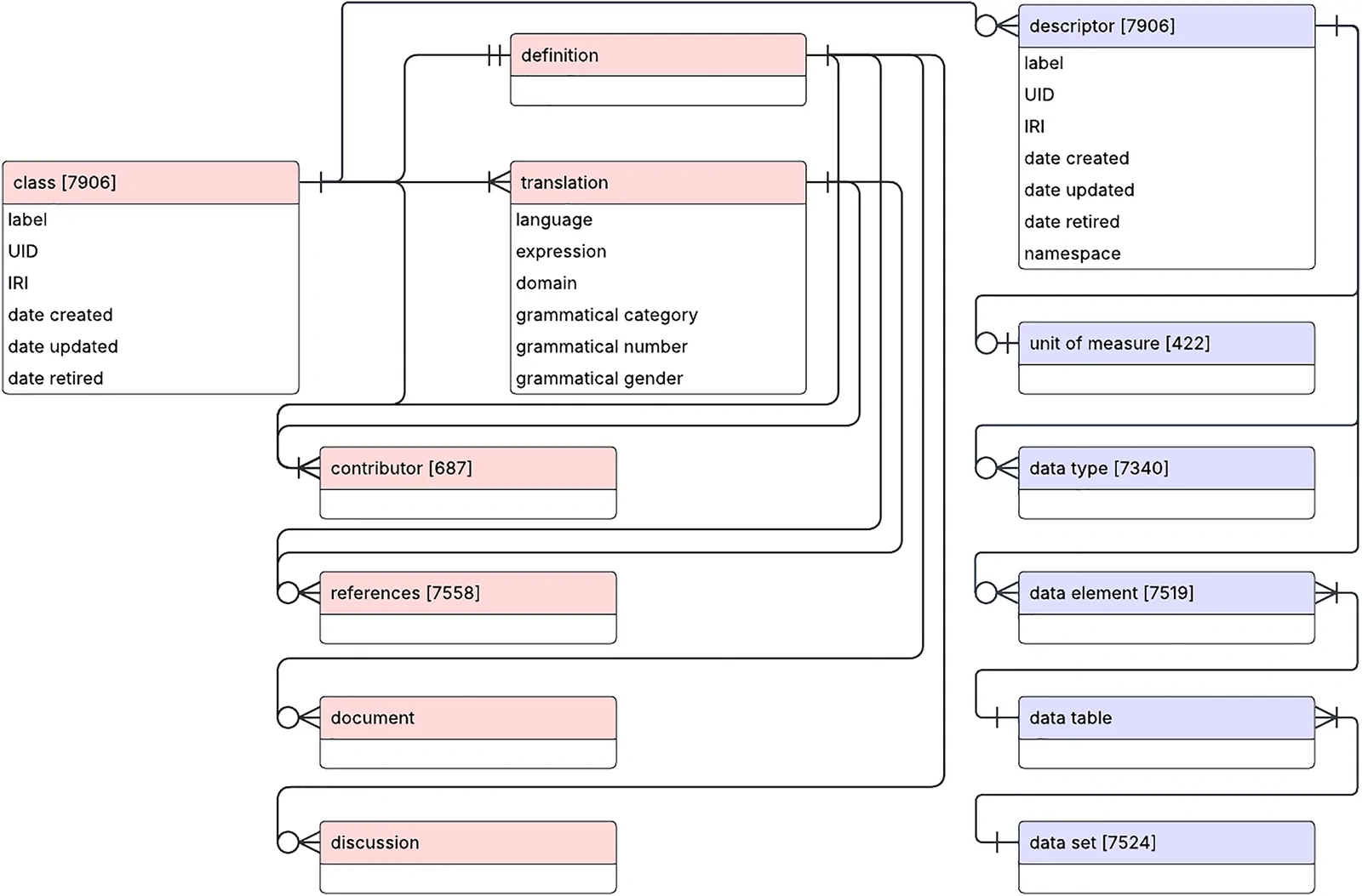
Simplified data model of the EUPH Metadata Collection. Red: EU Pollinator Hub Controlled Vocabulary (EUPH-CV) containing the Pollinator Ontology, Blue: EU Pollinator Hub metadata standard (EUPH-MS). Numbers in square brackets are the UID of the class assigned in the EUPH-CV. For some entities (class, translation, descriptor), their properties are included in the diagram. Cardinality and ordinality of relationships between entities are expressed by the following symbols at the starting and endpoint of each connecting line: vertical bar: one; two vertical bars: exactly one; zero and crow’s foot: zero or many; vertical bar and crow’s foot: one or many.

To facilitate the collection and sharing of data related to pollinators, the European Food Safety Authority (EFSA) sponsored the development of the EU Pollinator Hub (EUPH) [6], a web-based open-source software application [7] openly accessible at https://app.pollinatorhub.eu, which has been designed to enable standardisation and internationalisation of data related to pollinators, to ensure compliance with FAIR guiding principles for scientific data management and stewardship [8], and to facilitate interoperability of data formats and sharing of data. This is achieved both at the software level by various data and metadata management tools and at the procedural level by a set of openly accessible standard operating procedures (SOPs) and work instructions (WIs) [9]. An essential component of the EU Pollinator Hub is the EUPH Metadata Collection (Fig 1), a repository comprising the Controlled Vocabulary (EUPH-CV) – which contains the Pollinator Ontology (PolOn) – and the Metadata Standard (EUPH-MS).

The EUPH-MS is a stable standard reference for data providers to describe the data they intend to archive on the EU Pollinator Hub, comprising a set of unique metadata standard elements known as *descriptors*. A descriptor describes the meaning, unit and format of data. Descriptors have properties, including an associated class from the EUPH-CV that provides the descriptor’s definition. The EUPH-MS contains newly created elements and elements obtained from existing collections. Newly created elements include those issued by the Apimondia working group *Standardization of data on bees and beekeeping* [4] and by the EU Pollinator Hub development team [6]. They are referred to as the *Pollinator Metadata Standard* (PMS) [10]. Elements obtained from existing standards include a subset of elements from the Darwin Core metadata standard (DwC) and the Dublin Core Metadata Initiative (DCMI) elements contained therein [11] and elements from international and supranational standardising organisations such as the International Organization for Standardization (ISO) [12–15], the United Nations Statistics Division (UNSD) [16], Food and Agriculture Organization Corporate Statistical Database (FAOSTAT) [17], and EU institutions such as EFSA [18], the European Environment Agency (EEA) [19], and the European Statistical Office (Eurostat) [20] as well as some EU Regulations [21,22].

The EUPH-CV, the basis for the Pollinator Ontology, consists of building blocks referred to as *classes*. A class is a group of material or immaterial real-world entities (continuants such as information content, objects, organisms and parts thereof, and occurrents such as processes and temporal regions) that are sufficiently similar to be considered different from other classes in a given context. Classes have properties, such as a textual definition in the English language (including images and text documents as well as standardised references to other resources such as scientific literature or web resources), translations to human languages (to be considered as synonyms), a label, a unique identifier (UID), an internationalized resource identifier (IRI), dates of creation, update and deletion, a list of contributors, and a related descriptor from the EUPH-MS. Classes may also form nodes that are linked to other classes through relations. Relations to different classes are described using terms from knowledge representation languages, such as the Web Ontology Language (OWL) [23] and relationship ontologies, such as the Open Biomedical Ontologies (OBO) Relations Ontology (RO) [24], parts of which have been integrated into the EUPH-CV. OWL has been used to describe fundamental relations between classes, while RO has been used to describe domain-specific relationship types. Relations between classes are defined at the software level, where a set of relations to existing classes can be set when a class is created or updated, and at the procedural level, *i.e.,* in the construction of a textual definition of a class, where managers of the standardisation process are encouraged to reference terms and syntactic operators with hyperlinks to existing classes in the EUPH-CV. The latter should help human readers to interpret the definition correctly. The classes and the relationships between them serve as a backbone (1) for the definition of the metadata standard elements (EUPH-MS), (2) for the annotation of data stored on the EUPH in the form of literals or resource identifiers, (3) for the alignment of terminologies from different vocabularies, and (4) as a knowledge base for everyone. This should greatly facilitate the future analysis and processing of pollinator-related data.

A subset of the EUPH-CV has been designed as an ontology, the Pollinator Ontology (PolOn). Ontologies are defined as “*formal, explicit and shared representations of knowledge within a domain”* [25,26]. In practice, ontologies are controlled vocabularies in which terms are arranged in a hierarchical structure enriched with definitions, formal relationships between terms, and other properties (*e.g.*, relations to other terms, comments, images, and version information). They are a crucial technology in knowledge management, big data processing, and machine learning [25,27]. The use of terms from ontologies to annotate research data greatly improves automated data interpretation and interoperability [28] and, hence, compliance with FAIR principles [8].

The initial scope of the Pollinator Ontology was to standardise and internationalise terms related to pollinators and facilitate the integration of experimental or modelling data into the risk assessment and risk management domain. The main field of interest of the Pollinator Ontology is therefore the formal representation of knowledge about pollinators and their relationship with their abiotic and biotic environment, in particular with humans. The interaction with humans includes the economic exploitation of pollinators (in particular of the genus *Apis*), legislation (directly or indirectly) related to pollinators, concepts of veterinary medicine related to pollinators (currently restricted to *Apis mellifera*), the effect of plant protection products on pollinators, and, in particular, scientific studies related to pollinators. These interactions made it necessary to extend the Pollinator Ontology from the field of ecology to other domains, including agriculture (*e.g.,* beekeeping, breeding, production and trade of beekeeping products and services), veterinary medicine (*e.g.,* animal health management, active ingredients of veterinary medicinal products and diseases), chemistry (*e.g.,* chemical entities occurring in hive products or used as plant protection products), geography (*e.g.,* locations and spatial reference systems), economics (*e.g.,* macroeconomic parameters), sociology (*e.g.,* legal resources), and information science (*e.g.,* data items such as units of measurement and statistics). The above definition highlights that, unlike many other ontologies, it crosses a wide spectrum of domains with heterogeneous properties, reflecting the cross-domain nature of the subject, but it also sets its boundaries. The granularity of the presentation depends on user requirements, particularly the direction research on pollinators will take in the future.

A substantial part of the ontology consists of terms related to beekeeping, a domain which, to our knowledge, has not been covered by any openly accessible ontology. This has posed a particular challenge: the ontology had to be accessible to scientists and all types of operators in the beekeeping domain. In contrast to scientists, field operators may have limited language skills, necessitating the inclusion of translations of terms that allow both humans and machines to understand data, even if it is provided in a language other than English.

The Pollinator Ontology is openly available and can be accessed with any web browser without constraint. A version control system exists that avoids redistributing the entire ontology and single classes in altered form under the same properties. The creation and maintenance of the ontology have been designed to be collaborative and inclusive at both the process and software levels, allowing registered users of all backgrounds and educational levels to actively participate in class definition and translation.

The EUPH-CV, at the time of this manuscript’s submission, contained 8.232 classes and 30.773 translations to 88 languages. Of these, 2.688 classes (33%) with 12.655 translations (41%) form the Pollinator Ontology. It has been made available to the community under a CC BY 4.0 as a first version for revision and improvement. The process will be managed by the organisation BeeLife in the coming years.

## Materials and methods

Analysis of the Pollinator Ontology was performed in Python (version 3.13) in a JupyterLab notebook (Version 4.3.4). The data used for the analysis, including the computational document, can be found on Zenodo [29] and on the EUPH at https://app.pollinatorhub.eu/dataset-discovery/PLLNT287.0.0. The ontology has been made available on GitHub (https://gitlab.com/bee-life/pollinator-hub) in the Web Ontology Language (OWL) using the OWL 2 EL profile and EL++ expressivity.

**Fig 2:**
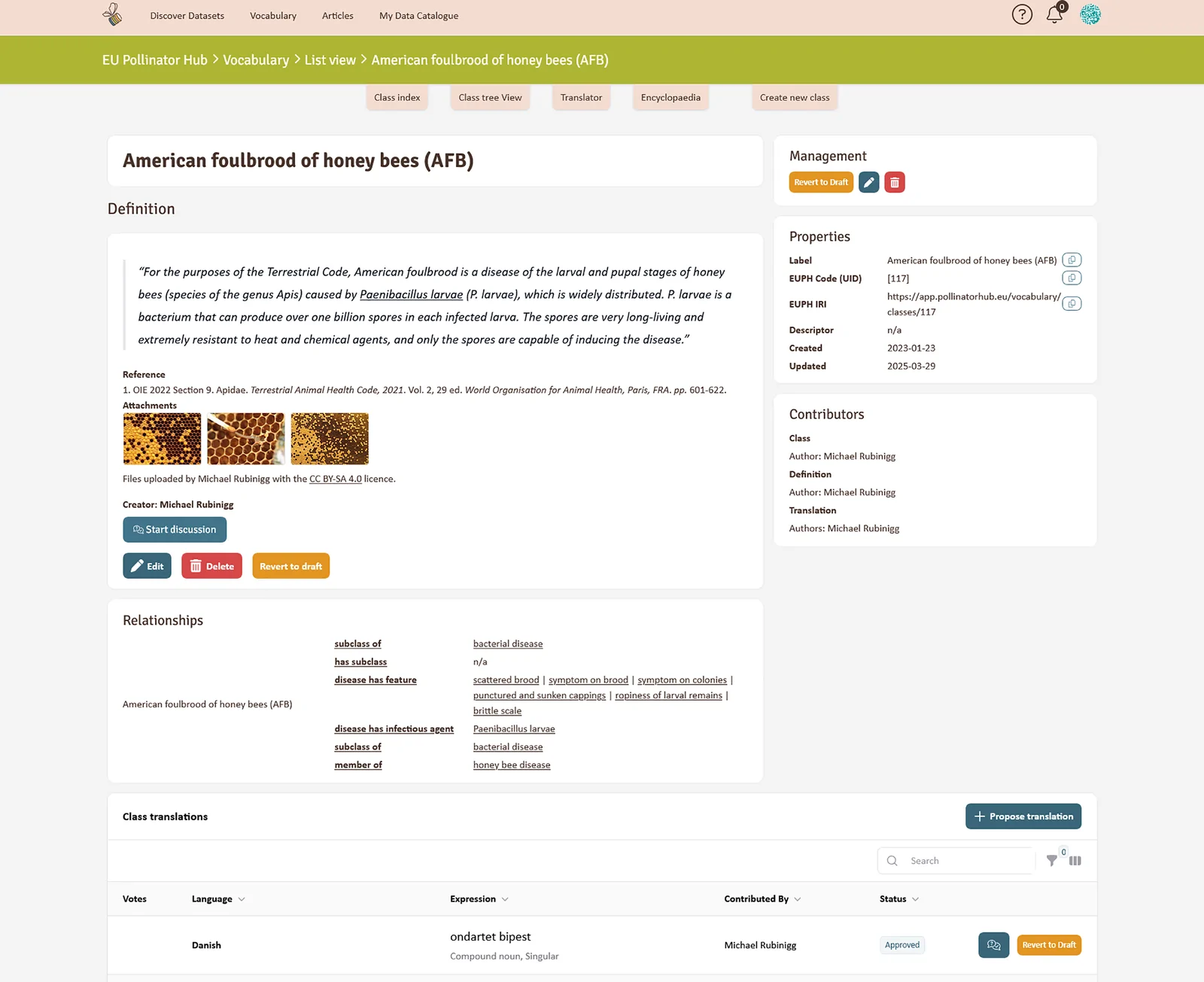
User interface of the EU Pollinator Hub for the management of the EUPH Controlled Vocabulary (https://app.pollinatorhub.eu/vocabulary/classes/117) showing all controls required for the management of a class. Explanations see text.

The components of the EU Pollinator Hub (https://app.pollinatorhub.eu) that are dedicated to the creation and maintenance of the ontology are the software application itself, accessible at https://app.pollinatorhub.eu/vocabulary (Fig 2), and a set of SOPs and WIs, regulating the process of metadata life cycle management on the EU Pollinator Hub, as well as conventions and rules for naming, accessible at https://app.pollinatorhub.eu/pages/sop-directory. Instructions for using the interface are available in the EUPH product directory (https://app.pollinatorhub.eu/pages/documentation). The management of the ontology is regulated in the EUPH SOP 012 (Data standardisation) [9] and processed with the EUPH software application (Fig 2).

In the first step of the workflow, a qualified process manager with the necessary access rights, appointed by the manager of the EU Pollinator Hub, creates a class, decides whether this class is part of the ontology and adds a unique label (a name in human language) to the class according to a predefined set of rules regarding, amongst others, language and orthography. The label’s uniqueness is validated by the software, which prevents homonymy. Subsequently, the process manager assigns relations to the class. Classes must at least be assigned to a superclass using the relation *subclass of* [23]. The assignment of a superclass, as defined in SOP 012, determines the structure of the controlled vocabulary. In addition to this basic relationship, an unlimited number of functional relations can be added by selecting a relationship from a preconfigured list that contains all relationships imported into the EUPH-CV, mainly from the OBO Relation Ontology (RO) [30].

Due to the cross-domain nature of the topic, the Pollinator Ontology inevitably overlaps with other ontologies. In such cases, an existing class from another ontology is imported by adding the IRI of this class as a class property. The decision on whether to adopt a class from another ontology is regulated in SOP 012 in general terms (relevance to the respective purpose; frequency of use; compliance with FAIR principles [8]) and shall be left to the process manager.

Once the process manager has created the class, the system assigns a UID, an IRI, a creation date, and an author. The UID, also referred to as the EUPH code, is a semantically free incrementing integer. If no specific IRI has been defined, an IRI on the EU Pollinator Hub is created, which is composed of the locator https://w3id.org/euph/ and the EUPH code of the respective class.

In the second step of the workflow, the process manager, alone or with registered users, provides a definition in English. Definitions should include a reference and may contain additional information assets such as text documents or images. In the case of imported classes, the definition of these classes has usually been used, clearly marked as citations and referenced. Once the definition has been created, the system adds all registered users who have contributed to the discussion thread as contributors to the definition. From this point onwards, registered users may interact with the process through an integrated messenger function. All final decisions rest with the process manager.

In the third step, translations into one or more human languages are provided, enabling both data annotation and data retrieval across language barriers. Translations carry grammatical properties and may be domain-specific. The process is governed by the relevant work instructions at https://app.pollinatorhub.eu/pages/sop-directory.

The life cycle of all metadata assets is regulated in EUPH SOP 016 for Release Management [9]. Newly created classes receive a preliminary status, which is consolidated after the deployment of a new ontology release, following approval by an administrator who supervises the process. Process managers cannot make changes to consolidated classes, except for deprecating them. When critical changes to a class are needed that may affect other data assets, classes must first be deprecated. Deprecated classes are not removed, but become a subclass of the class *obsolete class* (EUPH code 3281).

All versions of the Pollinator Ontology are published under a Creative Commons Attribution 4.0 license and are available at the software repository [7] and Zenodo [31].

All classes were created during data integration in the EU Pollinator Hub’s final development phase. The prioritisation of entities to be represented in the ontology was therefore based primarily on scientific and economic practice rather than a theoretical concept. Ethical approval was not required for this study, as it did not involve human participants or animals.

## Results

**Fig 3:**
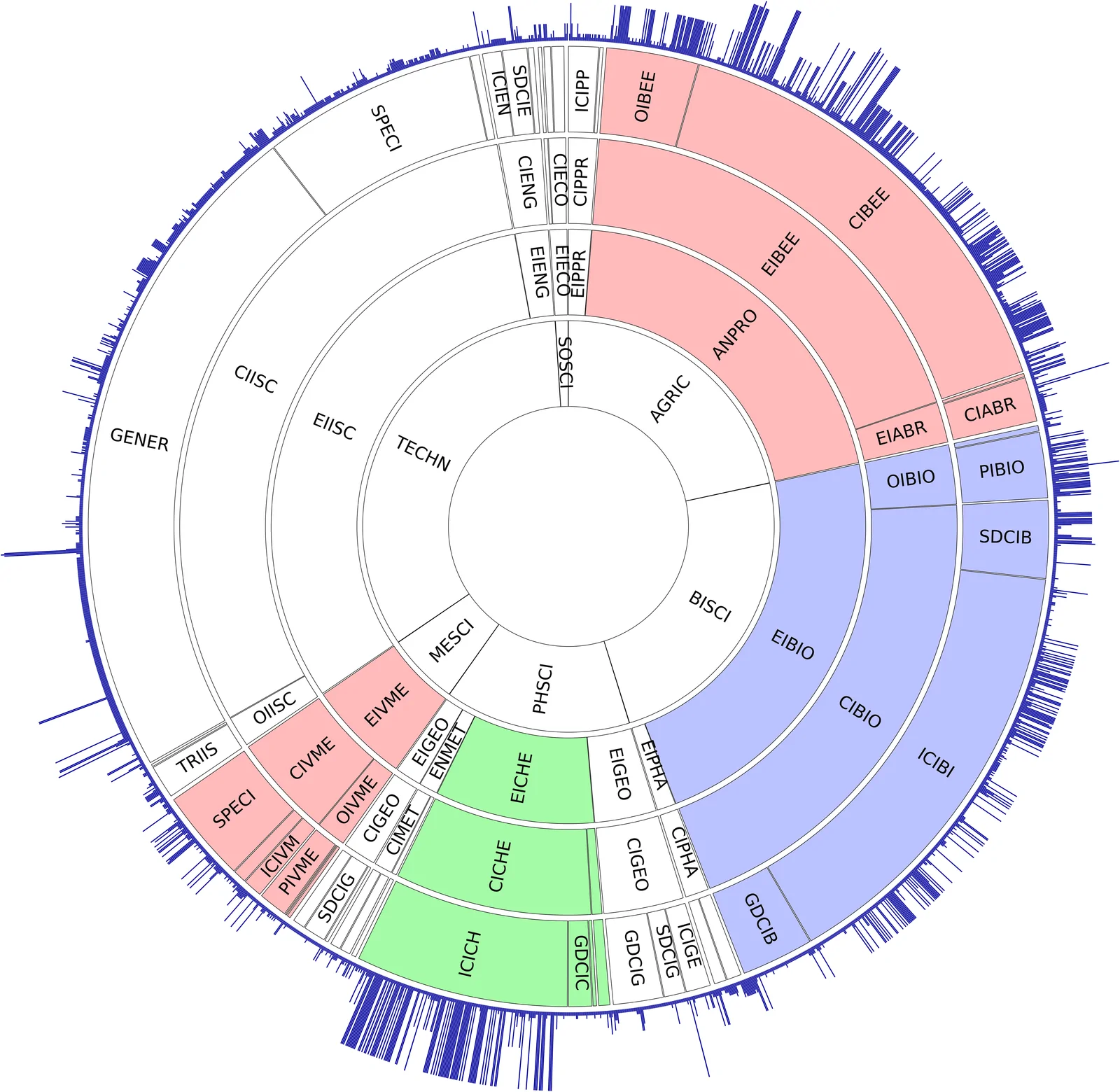
Graphical representation of the Pollinator Ontology. Terms related to the biology of pollinators (light blue), beekeeping (light red) and chemical entities (light green). Dark blue bars in the outer track of the circle represent the number of languages in which the terms have been translated. Each character quadruplet represents the name of a node (see text).

Of the 2.688 classes currently contained in the Pollinator Ontology (Table 1, Fig 3), 19% are related to beekeeping in the strict sense (subclasses of the node class *beekeeping*, EIBEE) and 26% to beekeeping in the broad sense, including subclasses of the root node *animal breeding* (EIABR) and *veterinary medicine* (EIVME). Another 31% are related to the domains *biology* (EIBIO) and *chemistry* (EICHE). A substantial part of the classes (32%) is associated with the domain *information science* (EIISC), which contains terms related to data and metadata assets, among which collections relevant for the operation of the EU Pollinator Hub (*e.g.,* Darwin Core, International Organization for Standardization, Bureau International des Poids et Mesures).

**Table 1:** Summary of the domains in the Pollinator Ontology and the number and percentage (in brackets) of the subclasses.

First-level domain	Second and higher-level domains			Number and percentage of subclasses
biological sciences	entity in pharmacology			27 (1,0%)
	entity in biology			603 (22,6%)
medical sciences	veterinary medicine			150 (5,6%)
physical sciences	entity in meteorology			27 (1,0%)
	entity in geology			38 (1,4%)
	entity in geography			90 (3,4%)
	entity in chemistry			232 (8,7%)
social sciences	entity in economics			25 (0,9%)
technology	agriculture	entity in plant production		33 (1,2%)
		animal production	entity in animal breeding	45 (1,7%)
			entity in beekeeping	497 (18,8%)
	entity in information science			847 (31,8%)
	entity in engineering			48 (1,8%)
TOTAL				2.688 (100%)

Note that some classes may appear in more than one domain and that first, second, and higher-level domains are not included in the total number of subclasses.

A total of 781 classes have been imported from other ontologies or controlled vocabularies (the number of classes is given in brackets). The most abundant sources were ontologies from Open Biological and Biomedical Ontologies (510) and the Global Biodiversity Information Facility (GBIF, 210). Among the former, the most frequently used ontologies include the National Cancer Institute thesaurus (NCIt) OBO Edition for most domain terms and terms related to biological analysis [32], the Hymenoptera Anatomy Ontology (HAO) in combination with the Uberon multi-species anatomy ontology [33,34] for anatomical entities, the Units of measurement ontology (UO) for units of measure and quantities [35], the Statistical Ontology (STATO) for terms related to mathematical and statistical analyses [36], the Environment Ontology for environmental entities [37], the Chemical Entities of Biological Interest Ontology (ChEBI) for chemical entities [38], as well as some minor ontologies from the agricultural domain. A collection of ontologies from which the Pollinator Ontology has reused classes and properties can be found under the class *reference ontology* (EUPH code 7752). Other ontologies, in particular from the agricultural domain, such as the Animal Health Ontology for livestock (AHOL, 5), the Animal Trait Ontology for livestock (ATOL, 6), and the INRAE Thesaurus (INRAEThes, 3), were used to a minor extent.

An essential property of classes that confer meaning to humans is the translation into human language. The Pollinator Ontology currently contains 12.655 translations into 88 languages. The five most common languages in the Pollinator Ontology are English (24,4% of all translations), German (15,0%), Dutch (6,5%), French (6,3%), and Italian (6,3%). The Romance languages Portuguese, Spanish and Romanian make up 15,8%, the Scandinavian languages Swedish, Danish and Norwegian make up 12,6% and other languages make up 13,0% of the translations.

Another essential property of classes conferring meaning to both humans and machines is their relationship with other classes (Table 2). Currently, 4.472 relationships have been defined for the Pollinator Ontology. The most abundant relationships (number of occurrences are given in brackets) between classes are *subclass of* (2.695) [39], which is a basic relationship between classes, and *member of* (607) [24], which usually describes the relationship between a class and a collection which it is part of, followed by *quality of* (174). Minor relationship types are *is unit of* (89), *part of* (86), *involved in* (74) and *provides nutrients for* (50) [24]. A summary of relationships with occurrences greater than 25 is given in Table 2.

**Table 2:** Summary of the relationships between classes in the Pollinator Ontology with more than 25 objects.

EUPH label	IRI	Number
subclass of	https://www.w3.org/2000/01/rdf-schema#subClassOf	2.695
member of	http://purl.obolibrary.org/obo/RO_0002350	607 (34,2%)
quality of	http://purl.obolibrary.org/obo/RO_0000080	174 (9,8%)
is unit of	http://semanticscience.org/resource/SIO_000222	89 (5,0%)
part of	http://purl.obolibrary.org/obo/BFO_0000050	86 (4,8%)
involved in	http://purl.obolibrary.org/obo/RO_0002331	74 (4,2%)
provides nutrients for	http://purl.obolibrary.org/obo/RO_0002469	50 (2,8%)
has role	http://purl.obolibrary.org/obo/RO_0000087	41 (2,3%)
has part	http://purl.obolibrary.org/obo/RO_0000051	34 (1,9%)
has flowers visited by	http://purl.obolibrary.org/obo/RO_0002623	29 (1,6%)
derives from part of	http://purl.obolibrary.org/obo/ENVO_0100300	29 (1,6%)
role of	http://purl.obolibrary.org/obo/RO_0000081	28 (1,6%)
has output	http://purl.obolibrary.org/obo/RO_0002234	28 (1,6%)
towards	http://purl.obolibrary.org/obo/RO_0002503	26 (1,5%)
produced by	http://purl.obolibrary.org/obo/RO_0003001	26 (1,5%)
pollinated by	http://purl.obolibrary.org/obo/RO_0002456	26 (1,5%)
Rest		430 (24,2%)
Relationships without *subclass of*		1.777 (100,0%)
**TOTAL**		**4.472**

EUPH label: name of the relationship at the EU Pollinator Hub; IRI: Internationalised Resource Identifier of the relationship; Number: Number of relationships between classes in the Pollinator Ontology (percentage in brackets refers to the number of axioms without the *subclass of* relationship).

## Discussion

The Pollinator Ontology uses BFO as an upper ontology. Formal relations are expressed using OWL and the OBO Relations Ontology (RO); domain-specific relationships are encoded using RO properties. Imported classes were mainly reused from NCIt (most domain terms and biological analysis terms), HAO and Uberon (anatomy), UO (units of measure), STATO (statistics), ENVO (environment), and ChEBI (chemical entities); the full list of reference ontologies is accessible under class reference ontology (EUPH code 7752) at https://app.pollinatorhub.eu/vocabulary.

The Pollinator Ontology, a subset of the EU Pollinator Hub Controlled Vocabulary, has been developed to improve the standardisation and internationalisation of pollinator data across all domains relevant to describing interactions between pollinators and their biotic and abiotic environment, in particular with humans. It addresses a domain that existing ontologies do not cover, neither in terms of a fully operational vocabulary nor in terms of the cross-domain integration required to support risk-assessment-aligned workflows for pollinators. To our knowledge, this is the first ontology that describes terms specifically related to pollinators, except for the Plant-Pollinator Interactions Controlled Vocabulary (PIP) [40]. PIP, however, offers only a limited set of terms (53) in a format similar to Darwin Core, a metadata standard rather than an ontology. Apart from this confusion, the IRI of PIP is currently not fully accessible, which is why it has been decided not to integrate it in the Pollinator Ontology.

Many ontologies represent aspects relevant to pollinators, but none cover all the production, veterinary, legal, or economic dimensions of pollinator science. The Hymenoptera Anatomy Ontology (HAO) and Uberon multi-species anatomy ontology [33,34] contain a vast number of anatomical terms but lack specificity for the group Apoidea and other pollinators. This situation has been improved, at least to some extent, in the Pollinator Ontology; the foundations have been laid for future work in this area. The Environment Ontology (ENVO) [37] provides environmental entities but lacks pollinator-specific operational context, a gap that the Pollinator Ontology addresses. Darwin Core (DwC) provides an important metadata standard for biodiversity observations, but it is not an ontology. Where possible, the Pollinator Ontology has been aligned with Darwin Core entities to ensure interoperability with biodiversity data. Integration of terms from the Chemical Entities of Biological Interest Ontology (ChEBI) [38] and the Gene Ontology (GO) [41] has, for the first time, enabled modelling of interactions between insecticide active ingredients and metabolic pathways in insect pollinators.

Most importantly, the Pollinator Ontology is the first ontology to incorporate a very large set of terms (currently circa 500) related to beekeeping. Although some classes in the beekeeping domain appear in agricultural ontologies such as the Agronomy Ontology (AgrO) [42], none of these ontologies provide a comprehensive and systematic representation of honey bees and beekeeping. Thanks to the systematic classification of terms and their translation into various languages, this should provide valuable support for the beekeeping industry. Particularly noteworthy in this context is that a considerable part of Crane’s extensive work, the Dictionary of Beekeeping Terms series [43–45], has been processed for the first time in a digital format. So far, 375 of approximately 1.000 terms related to bees and beekeeping, including their translations, have been included from this standard reference work published by Crane and co-workers.

The 88-language translation layer is a further differentiator: it enables field operators to annotate data in their native language, a requirement with no equivalent in any existing pollinator-related vocabulary.

Governance of the vocabulary currently operates through community practice and the standard operating procedures and work instructions publicly accessible at https://app.pollinatorhub.eu/pages/sop-directory. Formalisation of governance through dedicated metadata lifecycle management procedures is planned as the next development step.

Discoverability and long-term accessibility are addressed through several mechanisms already operational: the vocabulary is browsable without login at https://app.pollinatorhub.eu/vocabulary; all versions are archived under CC BY 4.0 on Zenodo (https://doi.org/10.5281/zenodo.18005994) and on GitLab (https://gitlab.com/bee-life/pollinator-hub); the EUPH API (https://app.pollinatorhub.eu/api/documentation) enables programmatic access to all classes, properties, translations, and relationships; and a community contribution interface allows registered users to participate in class definition and translation. The ontology has been submitted to the OBO Foundry. Indexing in OLS4 and BioPortal is planned pending approval by the OBO Foundry.

The decision to divide the ontology into domains (agriculture, biological sciences, medical sciences, physical sciences, social sciences, technology) and subdomains was primarily motivated by the need to ensure a consistent view within each domain, in accordance with the general principle of perspectivism [46]. For example, while a colony (EUPH code 6), defined as “a group of two or more organisms living in close association with, or connected to, one another” from a biologist’s perspective, is an object aggregate in the context of BFO, from a beekeeper’s management perspective, it might make more sense to classify it as an object. Another reason why this decision was taken is the fact that pollinators, more than any other organisms, ecologically or economically interact with a high number of very distinct domains (*e.g.,* plant biology, economy, agronomy, law, *etc.*), which is uncommon for ontologies, given that they are usually designed to cover specific subject matters. If this approach does not gain acceptance, steps have been taken to ensure that the domains can be merged without significantly affecting the properties of the classes.

The reasons for using a native user interface for the development of the Pollinator Ontology instead of relying on a proprietary developer platform such as GitLab or GitHub or dedicated software such as Protégé were (1) the need for fast and flexible management of metadata assets for the integration of data on the EUPH, in particular during the development phase, (2) the resulting opportunity to provide an inclusive tool with easy access to domain experts of all educational backgrounds, and (3) the availability of additional features that facilitate the management of the vocabulary (*e.g.,* tracking of authorship, assignment of relationships, edit history). Besides, the fact that the Pollinator Ontology, unlike other ontologies, has been designed for use and continuous extension within the same environment in which it is published (the EU Pollinator Hub) should significantly improve its accessibility and promote its use and dissemination. Despite the community-oriented approach, care has been taken at the procedural and software levels to ensure that the class lifecycle and conflict resolution are carried out by qualified individuals selected and explicitly authorised by the platform administrator.

As noted earlier, we integrated terms from existing ontologies, especially in root classes and nodes. The decision on which ontology to use was based on domain specificity and the extent to which a subset of terms of an existing ontology covered the set of terms of a specific subdomain in the Pollinator Ontology. For example, root terms for biological classification were adopted from the National Cancer Institute thesaurus (NCIt), which provided consistent coverage of biological hierarchies; anatomical root terms were sourced from Uberon and HAO, which had already been rigorously reviewed for Hymenoptera; and unit terms were taken from UO, which is widely used across OBO-compliant ontologies, thereby maximising interoperability with other life science data resources. Where partial overlap between existing ontologies could not be avoided, the more domain-specific ontology was preferred as the canonical source, in line with the orthogonality principle of the OBO Foundry [30].

The first dataset in which the ontology has been used as a proof of concept was *Pollination* [47], also available in a thoroughly documented version at https://app.pollinatorhub.eu/dataset-discovery/PLLNT11.0.0. It contains data on the economic dependence of pollination by animals in crops, as well as observations of pollinators on plant species, particularly crops. To illustrate how the ontology supports FAIR data annotation in practice, consider the descriptor Varroa infestation rate of adult bees (pms:varroaInfestationOfAdultBees, UID 0.0.VRRNF468, IRI https://app.pollinatorhub.eu/vocabulary/descriptors/0.0.VRRNF468). Its associated CV class (EUPH code 7348, IRI https://app.pollinatorhub.eu/vocabulary/classes/7348) provides a formal definition anchored to the COLOSS Beebook alcohol-wash protocol [48], with a unit of Varroa mites per 100 adult bees. As of submission, three datasets comprising 3,681 data points have been deposited against this descriptor. The descriptor is Findable via its persistent IRI; Accessible through the open EUPH API (https://app.pollinatorhub.eu/api/documentation); Interoperable because data annotated with its IRI can be unambiguously joined across datasets from different providers; and Reusable because the class definition, unit, and measurement protocol are machine-readable and unambiguous. This example demonstrates that the Pollinator Ontology is not merely a classification scheme but a functioning annotation infrastructure with real datasets.

Except for some fields (*e.g.,* genomic research), data standardisation and accessibility in life sciences in general [49] and in pollinator research in particular (despite various efforts [33,49–51]), but especially in the beekeeping sector [3–5], require optimisation. The EU Pollinator Hub provides all the necessary tools for community-driven development of the Pollinator Ontology at the procedural and software levels, including a version control system and tools for visualisation, reporting, exploration, and collaboration. It has been designed to comply with the rules set by the Open Biological and Biomedical Ontology Foundry [30], the guidelines for minimum information for the reporting of an ontology (MIRO) [52] and the FAIR guiding principles for scientific data management and stewardship [8]. Beyond the scientific and technical significance of the ontology, the publicly accessible part of the application for exploring the controlled vocabulary provides a valuable tool for all users to access concise, accurate information, translations, and additional resources on pollinators. Data providers on the EU Pollinator Hub and beyond are encouraged to use the controlled vocabulary to code data for machine processing using the terms’ IRIs and other properties (UIDs, relationships, translations).

The first version of the Pollinator Ontology, which has only recently been deployed in Zenodo [29,31] and submitted to the OBO foundry, already contains a large number of elements, which are supposed to initiate the process of standardisation and internationalisation of data in the domain of pollinator research, pollinator protection and the economic exploitation of pollinators. Scientists, environmental risk assessors and risk managers, policymakers, veterinarians, technicians and beekeepers are supposed to be the adopters and drivers of this process, accessible through the EU Pollinator Hub, a web-based platform sponsored by the European Food Safety Authority (EFSA) and currently maintained by the European NGO BeeLife European Beekeeping Coordination. In the future, the Pollinator Ontology should be developed by the Apimondia working group *Standardization of data on bees and beekeeping* [4]

## Conclusion

The development of the Pollinator Ontology represents a major step toward standardising and internationalising pollinator-related data across disciplines. The inclusion of beekeeping-specific terminology, multilingual support, and its integration within the EU Pollinator Hub ensures both scientific robustness and practical utility. Future efforts will focus on community engagement, iterative refinement of terms, and alignment with emerging data standards to foster widespread adoption across research, policy, and operational contexts.
