# Supplementary material for: The EU pollinator hub controlled vocabulary: An ontology for pollinators
Source: PLoS One. 2026 Aug 26;21(8):e0353382. doi: 10.1371/journal.pone.0353382 (PMC13514042; doi:10.1371/journal.pone.0353382)
Supplement: Supplementary file 1 [file pone.0353382.s003.pdf]

REVIEW OF RUBINIGG ET AL

1. Please ensure that your manuscript meets **PLOS ONE's style requirements,** including those for file naming. The PLOS ONE style templates can be found at

https://journals.plos.org/plosone/s/file?id=wjVg/PLOSOne_formatting_sample_main_body.pdf and

https://journals.plos.org/plosone/s/file?id=ba62/PLOSOne_formatting_sample_title_authors_affiliations.pdf

Style modifications have been performed to meet PLOS ONE's style requirements

2. We note that the grant information you provided in the **'Funding Information' and 'Financial Disclosure' sections do not match.**

When you resubmit, please ensure that you provide the correct grant numbers for the awards you received for your study in the 'Funding Information' section.

This was modified, thank you

3. Please note that funding information should not appear in any section or other areas of your manuscript. We will only publish funding information present in the Funding Statement section of the online submission form. Please **remove any funding-related text** from the manuscript.

This was removed, thank you

4. Thank you for uploading your study's underlying data set. Unfortunately, the **repository you noted in your Data Availability statement does not meet PLOS's standards** for an acceptable data repository.

At this time, please upload the minimal dataset necessary to replicate your study's findings to a stable, public repository (such as figshare or Dryad) and provide us with the relevant URLs, DOIs, or accession numbers that can be used to access these data. For a list of recommended repositories and additional information on PLOS standards for data deposition, please see https://journals.plos.org/plosone/s/recommended-repositories.

The data have been included in the Zenodo repository, in addition to the EU Pollinator Hub, and the links have been included in the Manuscript.

5. We note that your Data Availability Statement is currently as follows: " All relevant data are within the manuscript and its Supporting Information files. The EU Pollinator Hub Controlled Vocabulary (EUPH-CV) is openly accessible at https://app.pollinatorhub.eu/vocabulary.

Metadata and versioned releases of the ontology are available under a Creative Commons Attribution 4.0 license via the BeeLife repository at https://gitlab.com/bee-life/pollinator-hub.

Please confirm at this time whether or not your submission contains all the raw data required to replicate the results of your study. Authors must share the "minimal data set" for their submission. PLOS defines the minimal data set as the data required to replicate all study findings reported in the article, along with related metadata and methods (https://journals.plos.org/plosone/s/data-availability#loc-minimal-data-set-definition).

For example, authors should submit the following data:

- The values behind the means, standard deviations and other measures reported;

- The values used to build graphs;

- The points extracted from images for analysis.

Authors do not need to submit their entire dataset if only a portion of it was used in the reported study.

If your submission does not contain these data, please either upload them as Supporting Information files or deposit them to a stable, public repository and provide us with the relevant URLs, DOIs, or accession numbers. For a list of recommended repositories, please see https://journals.plos.org/plosone/s/recommended-repositories.

If there are ethical or legal restrictions on sharing a de-identified data set, please explain them in detail (e.g., data contain potentially sensitive information, data are owned by a third-party organization, etc.) and who has imposed them (e.g., an ethics committee). Please also provide contact information for a data access committee, ethics committee, or other institutional body to which data requests may be sent. If data are owned by a third party, please indicate how others may request data access.

The resubmission includes all raw data and links to the raw data needed to replicate the study's results. The dataset is entirely available in the provided links, which are included in the reviewed manuscript. The Data were made available at Zotero in addition to EUPH (see references in the Manuscript).

6. We notice that your **supplementary figures are uploaded with the file type 'Figure'. Please amend the file type to 'Supporting Information'**. Please ensure that each Supporting Information file has a legend listed in the manuscript after the references list.

There are no supplementary figures, so this comment may refer to the figures to be included in the main manuscript. Yet, we do not understand this comment, because we do not know which figures are referred to.

7. If the **reviewer comments** include a recommendation to **cite specific previously published works**, please review and evaluate these publications to determine whether they are relevant and should be cited. There is no requirement to cite these works unless the

editor has indicated otherwise.

Additional Editor Comments:

Please pay particular attention to the comments provided by Reviewer 2, as they offer important insights and suggest practical improvements. In particular, there is a need for enhanced documentation of the ontology's functionality and adoption strategy, emphasising the importance of using standards like MIRO for ontology reporting, and the necessity to demonstrate the ontology's value through specific examples of data integration. Additionally, technical concerns such as the discoverability of the ontology and improvements to the user interface should be addressed to ensure that the tool is effective and easily adoptable by the research community. By focusing on these areas, your revised manuscript will be better aligned with the publication criteria of PLOS ONE.

[Note: HTML markup is below. Please do not edit.]

Reviewer's Responses to Questions

Comments to the Author

1. Is the manuscript technically sound, and do the data support the conclusions?

The manuscript must describe a technically sound piece of scientific research with data that supports the conclusions. Experiments must have been conducted rigorously, with appropriate controls, replication, and sample sizes. The conclusions must be drawn appropriately based on the data presented.

Reviewer #1: Yes

Reviewer #2: No

Reviewer #3: Yes

---

2. Has the statistical analysis been performed appropriately and rigorously?

Reviewer #1: N/A

Reviewer #2: N/A

Reviewer #3: N/A

---

3. Have the authors made all data underlying the findings in their manuscript fully available?

The PLOS Data policy requires authors to make all data underlying the findings described in their manuscript fully available without restriction, with rare exception (please refer to the Data Availability Statement in the manuscript PDF file). The data should be provided as part of the manuscript or its supporting information, or deposited to a public repository. For example, in addition to summary statistics, the data points behind means, medians and variance measures should be available. If there are restrictions on publicly sharing data—e.g. participant privacy or use of data from a third party—those must be specified.

Reviewer #1: Yes

Reviewer #2: No

Reviewer #3: Yes

---

4. Is the manuscript presented in an intelligible fashion and written in standard English?

PLOS ONE does not copyedit accepted manuscripts, so the language in submitted articles must be clear, correct, and unambiguous. Any typographical or grammatical errors should be corrected at revision, so please note any specific errors here.

Reviewer #1: Yes

Reviewer #2: Yes

Reviewer #3: Yes

---

5. Review Comments to the Author

Please use the space provided to explain your answers to the questions above. You may also include additional comments for the author, including concerns about dual publication, research ethics, or publication ethics. (Please upload your review as an attachment if it exceeds 20,000 characters)

Reviewer #1: The manuscript demonstrates a thoughtful integration of empirical evidence and conceptual clarity, and with further refinement in structure and argument flow, it holds strong potential to make a meaningful contribution to the field.

Thank you!

Reviewer #2: In order to be accepted, this paper needs much more information about the ontology itself and how the ontology improves data standardization/integration. I would

recommend looking at the MIRO (minimum information for reporting an ontology) standard. I am not convinced that an ontology is the most appropriate knowledge modelling structure to use here. Perhaps a knowledge graph would be better? Specific suggestions below.

The MIRO standard has been fully implemented, and its implementation has been discussed in the manuscript. At the time of manuscript submission, the ontology was still underdeveloped in some respects, particularly regarding the actual purpose of ontologies. The work of the last few months has significantly improved this, and we hope that the reviewer will appreciate these efforts.

1. Is this ontology findable via OLS, BioPortal, or OBO Foundry? It will not help standardize data if it is not adopted and to be adopted people have to know it exists.

The ontology was self-published in the EU Pollinator Hub and presented in relevant fora (Apimondia, pollinator-related projects, etc.). It has also been submitted to OBO Foundry, and importing to ontology lookup services requires the creation of an OWL file, which has also been done.

2. I would like to see a specific example of data standardization/harmonization/integration that demonstrates the value of this ontology

We have included an example in the manuscript.

3. In Fig 2, it is not clear what someone would do to initiate an edit and what kinds of edits are possible. I am also not convinced that your interface is better than using GitHub for collaborative ontology development.

We have added a section in the manuscript to justify the use of a native user interface rather than a development platform such as GitHub, and we have added a link to the EUPH product directory.

4. Have you registered your namespace with w3id.org? Probably better than managing EUPH identifiers yourself.

Thank you for this suggestion, we did it. The merge was successful, three namespaces are now registered and pointing to the Pollinator Hub:

- https://w3id.org/euph links directly to repository,
- https://w3id.org/euph/{id} links relative to class id, for example, https://w3id.org/euph/3344 links to the class with code 3344)
- https://w3id.org/pms/{name} links relative to a descriptor with given name that uses the ontology. For example https://w3id.org/pms/hiveWeight will open a class with the name hiveWeight (currently not implemented on our side)

5. In Table 1, who made the decision of which classes were root and secondary? Also, are the agricultural classes reusing agricultural ontologies?

The construction of root trees as well as the assignment of classes to a domain are regulated in SOP 012, section 5.2.2.5 (Ontology design). An explanation has been added in the manuscript. Regarding agricultural ontologies, some agricultural domains have indeed been integrated into the EUPH-CV. A short note has been added to the manuscript. Unfortunately, ontologies in this domain are often poorly developed and incomplete, especially in beekeeping, which makes it difficult to reuse them to a greater extent and was one of the reasons for building this ontology, as stated in the manuscript.

6. I would recommend adhering to the domain division rule for ontologies. Use a knowledge graph to traverse domains.

Thank you for the suggestion. The knowledge graph to traverse domains will be included in the OWL file

7. Where is the ontology OWL file?

The reviewer refers to this source: https://www.w3.org/TR/owl2-syntax/

8. You don't want to make EUPH another data silo. The ontology is supposed to help avoid this. How would data from EUPH integrate with data from related knowledge bases, such as a phenology-focused data resource, for example?

The EUPH aims to create a data repository that enables validation of the FAIRness and Quality of the data uploaded there, and then pushes for the standardisation of the data uploaded to it (which is why the PMS was created and integrated with other internationally available standards such as Darwin Core, ISO, etc.). The data is linked to Zenodo (supported by CERN and ensuring long-term preservation), and we are working to link it to several Data Journals, so users can choose to have their data in multiple repositories. This increases data redundancy and resilience. A data silo is created when the available data is not FAIR and cannot be interoperated with or reused. In essence, the very creation of the EUPH is to avoid this, so we do not really understand how it could be another data silo. We have to better explain this in the discussion.

Reviewer #3: PollinatorHub is a necessary initiative and, at the same time, a huge project. As a reviewer, I agree with the authors of this publication that there is a great deal of diverse data on pollinating insects, but it is extremely fragmented, not standardised, and therefore of limited use to others.
My only minor suggestion to the authors would be to add an example procedure to the article, perhaps in the form of a diagram, on how to enter one's own scientific data into the PollinatorHub database. In other words, a practical guide for the scientist user. The article is written in very technical language, so an example would probably be very helpful for the readers of this article.

Thank you for the comments. This comment does not refer to the ontology but to the data upload. A guide is available while users are uploading data. Some SOPs refer to the data

upload process (namely, 6, 9, 8, 12, and 13), and video tutorials show the procedure (https://www.youtube.com/watch?v=YNTI_capc1k&list=PLGe_DfwrNqSJkSPyUfyKseGof71h1oD5f&index=4). We are also working on a user-friendly user guide showing the step-by-step process.

Indeed, it may not be evident to the users. We have improved its accessibility so that potential users find it easier to upload their data.

---

6. PLOS authors have the option to publish the peer review history of their article (what does this mean?). If published, this will include your full peer review and any attached files.

If you choose "no", your identity will remain anonymous but your review may still be made public.

Do you want your identity to be public for this peer review? For information about this choice, including consent withdrawal, please see our Privacy Policy.

Reviewer #1: **Yes:** Charles Fernando dos Santos

Reviewer #2: **Yes:** Anne E Thessen

Reviewer #3: No
