# Supplementary material for: The EU pollinator hub controlled vocabulary: An ontology for pollinators
Source: PLoS One. 2026 Aug 26;21(8):e0353382. doi: 10.1371/journal.pone.0353382 (PMC13514042; doi:10.1371/journal.pone.0353382)
Supplement: Supplementary file 2 [file pone.0353382.s002.docx]

The manuscript presents a substantial and timely effort to develop a pollinator-focused ontology and controlled vocabulary, but it still needs clearer articulation of its novelty, stronger demonstration of practical utility, and better separation between platform description and ontology reporting to fully meet article-level standards.

**Overall assessment**

- The work is important: an openly available ontology for pollinators and beekeeping, tightly linked to a metadata standard (PMS) and implemented within the EU Pollinator Hub, directly addresses well-known problems of fragmented, non-standardised pollinator data.
- The resource is already reasonably mature (thousands of classes, 80+ languages, reuse of multiple OBO and related ontologies, versioning and SOPs), and the authors now align the description with MIRO and FAIR principles, which is appropriate for PLOS ONE.
- However, the manuscript still reads partly as a technical project report for the whole EUPH ecosystem, with relatively limited, concrete evidence of how the ontology itself improves data integration in practice, and with some conceptual and structural choices that warrant clearer justification.

**Major points for critique**

**1. Novelty and positioning of the ontology**

The paper claims that this is the first ontology to explicitly cover pollinators and beekeeping at this scale, and contrasts it mainly with the PIP vocabulary. While that is plausible, the argument is underdeveloped.

- The manuscript should more explicitly position EUPH‑CV and the Pollinator Ontology relative to existing biodiversity, agricultural and environmental ontologies (e.g. ENVO, AEO, crop/pollination vocabularies), beyond a list of reused sources.
- The cross‑domain scope (ecology, law, economics, data standards, etc.) is presented as a strength, but it risks making the ontology conceptually diffuse. The justification for structuring primarily by “scientific domain” rather than by upper‑ontology categories is brief and could be expanded to show why this is not just expedient but conceptually sound for pollinator data.

In its current form, the manuscript somewhat underplays the conceptual design decisions and overemphasises descriptive metrics (counts of classes, translations, etc.).

**2. Focus and structure of the article**

The paper mixes three layers:

- The EU Pollinator Hub platform (data archive, SOPs, upload workflows).
- The EUPH Metadata Standard (PMS + DwC + ISO, etc.).
- The Pollinator Ontology (subset of EUPH‑CV).

This layering is intellectually coherent but makes the manuscript dense and occasionally hard to follow for readers primarily interested in the ontology.

- Consider restructuring so that the ontology is clearly central: a shorter context section on EUPH and PMS, then a dedicated core section on ontology design, sources, reuse strategy, domain coverage, and reasoning capabilities, followed by a separate section on implementation in EUPH and user‑facing tools.
- Some current content is quite implementation‑heavy (e.g. detailed SOP references, UI-level workflow) and could be summarised or moved to Supplementary Material, with the main text focusing more on knowledge‑engineering aspects and use cases.

**3. Practical demonstration of value for data integration**

Reviewer 2 already requested a “specific example of data standardization/harmonization/integration that demonstrates the value of this ontology,” and the authors indicate that they added Pollination as a proof-of-concept dataset.

- At present, the Pollination example is only briefly mentioned as a dataset annotated with the ontology; there is no concrete, step‑by‑step illustration of how ontology terms (classes, IRIs, relations) were used to harmonise heterogeneous fields, nor what kinds of queries or interoperability become possible as a result.
- For a research article, at least one worked example is needed: show a small subset of real pollinator datasets before and after mapping to EUPH‑CV (e.g. different field names and vocabularies for species, hive metrics, pesticide exposures), then demonstrate how ontology‑based annotation enables unified querying or cross‑study aggregation that was not feasible before.

Without such an example, many of the claims about improved standardisation and FAIRness remain somewhat aspirational.

**4. Ontology design choices and governance**

The ontology uses BFO as an upper ontology in newer sections and imports from multiple OBO and related ontologies, while also introducing “private classes” for source‑specific terms (e.g. FAOSTAT, EFSA) linked via equivalentClass.

- The paper notes that domain partitioning is by scientific field and that some areas still reflect an older “lexical” structure predating full ontology design. This hybrid status is realistic but should be explicitly described as a current limitation and future work item: which parts are fully BFO‑compliant, which are still legacy, and how migration will proceed.
- The introduction of private classes and local equivalents (e.g. FAOSTAT’s Bees vs EUPH honey bee colony) is understandable, but the strategy and potential for duplication/confusion need clearer explanation: how will users know which class to use when annotating new data, and how will changes in external vocabularies be managed over time?
- Governance and community processes are mentioned (BeeLife, Apimondia WG, SOP 012 and SOP 016), but the criteria for accepting new classes, resolving conflicts, or deprecating terms could be more concretely illustrated.

This is important if the authors want to claim MIRO compliance and long‑term sustainability.

**5. Discoverability, interoperability and risk of “another silo”**

The authors rightly emphasise w3id registration, OBO submission, and Zenodo/GitLab hosting. They also respond to concerns about EUPH becoming another silo by arguing that FAIR and standardised data, linked out to external repositories and journals, prevent this.

- It would strengthen the argument to explicitly describe how EUPH‑annotated data could be discovered and reused from outside the EUPH environment. For example, how would a phenology database or a global pollinator trait database programmatically discover relevant classes and map their own fields to EUPH‑CV IRIs?
- The current text still focuses mainly on what happens within EUPH; more emphasis on external machine‑actionable access (e.g. through OLS, BioPortal, APIs) and some concrete interoperability scenarios would make the “not a silo” claim more convincing.

**Minor and stylistic points**

- Some numerical descriptions (counts of classes, translations, relationships) are helpful but could be streamlined; the key message is coverage and balance across domains, not the exact numbers.
- There are occasional small inconsistencies (e.g. slightly different class counts in main text vs tracked‑changes portion) that should be checked for coherence.
- The English is generally clear, but a final editorial pass would help tighten long sentences and remove informal asides (e.g. “we do not really understand how it could be another data silo”).

**Suggested recommendation**

On balance, this is a valuable community resource and clearly within the scope of a methods/infrastructure paper, but it still requires more focused exposition and a stronger, concrete demonstration of added value for data integration.

I would recommend **major revision**, with the main priorities:

- Re‑centring the manuscript on the ontology itself (design, scope, reuse, reasoning, governance) rather than the broader platform.
- Providing at least one clear, worked example of ontology‑enabled data harmonisation/integration.
- Clarifying conceptual design choices (domain‑based structure, legacy vs BFO‑based sections, private vs global classes) and how they will be managed as the ontology evolves.
- Expanding on external discoverability and real‑world interoperability to address the “data silo” concern more convincingly.
