# Supplementary material for: The EU pollinator hub controlled vocabulary: An ontology for pollinators
Source: PLoS One. 2026 Aug 26;21(8):e0353382. doi: 10.1371/journal.pone.0353382 (PMC13514042; doi:10.1371/journal.pone.0353382)
Supplement: Supplementary file 3 [file pone.0353382.s004.docx]

Reviewers' comments:

Reviewer's Responses to Questions

**Comments to the Author**

1. If the authors have adequately addressed your comments raised in a previous round of review and you feel that this manuscript is now acceptable for publication, you may indicate that here to bypass the “Comments to the Author” section, enter your conflict of interest statement in the “Confidential to Editor” section, and submit your "Accept" recommendation.

Reviewer #4: All comments have been addressed

Reviewer #5: All comments have been addressed

2. Is the manuscript technically sound, and do the data support the conclusions?

The manuscript must describe a technically sound piece of scientific research with data that supports the conclusions. Experiments must have been conducted rigorously, with appropriate controls, replication, and sample sizes. The conclusions must be drawn appropriately based on the data presented.

Reviewer #4: Yes

Reviewer #5: Partly

3. Has the statistical analysis been performed appropriately and rigorously?

Reviewer #4: N/A

Reviewer #5: Yes

4. Have the authors made all data underlying the findings in their manuscript fully available?

The [PLOS Data policy](http://track.editorialmanager.com/CL0/http:%2F%2Fwww.plosone.org%2Fstatic%2Fpolicies.action%23sharing/1/010f019cdc0c4ad3-9cbc2002-310c-4ba3-b4f7-4b8964cb94dc-000000/nQbeBgbM5zl_97rX8mJh0Kr489grQOfgCGZTgDCwxJ8=253) requires authors to make all data underlying the findings described in their manuscript fully available without restriction, with rare exception (please refer to the Data Availability Statement in the manuscript PDF file). The data should be provided as part of the manuscript or its supporting information, or deposited to a public repository. For example, in addition to summary statistics, the data points behind means, medians and variance measures should be available. If there are restrictions on publicly sharing data—e.g. participant privacy or use of data from a third party—those must be specified.

Reviewer #4: Yes

Reviewer #5: Yes

5. Is the manuscript presented in an intelligible fashion and written in standard English?

PLOS ONE does not copyedit accepted manuscripts, so the language in submitted articles must be clear, correct, and unambiguous. Any typographical or grammatical errors should be corrected at revision, so please note any specific errors here.

Reviewer #4: Yes

Reviewer #5: No

**6. Review Comments to the Author**

Please use the space provided to explain your answers to the questions above. You may also include additional comments for the author, including concerns about dual publication, research ethics, or publication ethics. (Please upload your review as an attachment if it exceeds 20,000 characters)

Reviewer #4: I think the manuscript "The EU Pollinator Hub Controlled Vocabulary: An Ontology for Pollinators" is a valuable addition to achieve usability of the much-needed Pollinator Hub. I find that previous comments are answered and the manuscript is ready for consideration of accepting for publishing.

Thank you for your feedback

Reviewer #5: The manuscript presents a substantial and timely effort to develop a pollinator-focused ontology and controlled vocabulary, but it still needs clearer articulation of its novelty, stronger demonstration of practical utility, and better separation between platform description and ontology reporting to fully meet article-level standards.

Thank you for your feedback. We hope to have achieved this in this revision.

Overall assessment
• The work is important: an openly available ontology for pollinators and beekeeping, tightly linked to a metadata standard (PMS) and implemented within the EU Pollinator Hub, directly addresses well-known problems of fragmented, non-standardised pollinator data.
• The resource is already reasonably mature (thousands of classes, 80+ languages, reuse of multiple OBO and related ontologies, versioning and SOPs), and the authors now align the description with MIRO and FAIR principles, which is appropriate for PLOS ONE.
• However, the manuscript still reads partly as a technical project report for the whole EUPH ecosystem, with relatively limited, concrete evidence of how the ontology itself improves data integration in practice, and with some conceptual and structural choices that warrant clearer justification.

Thank you for the feedback. We included an extensive section in the Discussion that provides context for our development, giving the manuscript a broader perspective on the ontology's impact and utility.

Major points for critique
1. Novelty and positioning of the ontology
The paper claims that this is the first ontology to explicitly cover pollinators and beekeeping at this scale, and contrasts it mainly with the PIP vocabulary. While that is plausible, the argument is underdeveloped.
• The manuscript should more explicitly position EUPH CV and the Pollinator Ontology relative to existing biodiversity, agricultural and environmental ontologies (e.g. ENVO, AEO, crop/pollination vocabularies), beyond a list of reused sources.
• The cross domain scope (ecology, law, economics, data standards, etc.) is presented as a strength, but it risks making the ontology conceptually diffuse. The justification for structuring primarily by “scientific domain” rather than by upper ontology categories is brief and could be expanded to show why this is not just expedient but conceptually sound for pollinator data.
In its current form, the manuscript somewhat underplays the conceptual design decisions and overemphasises descriptive metrics (counts of classes, translations, etc.).

We thank the reviewer for this constructive critique. We agree that the positioning relative to broader biodiversity and agricultural ontologies was underdeveloped, and we have substantially expanded the Discussion to address this. A new paragraph has been added that explicitly contrasts the EUPH-CV with ENVO, Darwin Core, HAO/Uberon, and crop-specific vocabularies, and articulates the three complementary dimensions that distinguish the EUPH-CV: (1) its comprehensive, domain-spanning pollinator-specific scope; (2) its multilingual layer across 88 languages; and (3) its formalisation of the beekeeping controlled vocabulary, previously existing only in specialist print dictionaries. We also clarify that the cross-domain scope is not conceptually diffuse, but rather a necessary consequence of the ecological and regulatory position of pollinators: unlike narrowly defined biological taxa, pollinators are simultaneously objects of ecological research, veterinary management, agricultural regulation, economic valuation and international policy. An ontology that does not span these domains cannot support the data integration use cases for which it was designed. Tracked changes are visible in the revised manuscript. We hope to have addressed the reviewer's comments.

2. Focus and structure of the article
The paper mixes three layers:
• The EU Pollinator Hub platform (data archive, SOPs, upload workflows).
• The EUPH Metadata Standard (PMS + DwC + ISO, etc.).
• The Pollinator Ontology (subset of EUPH CV).
This layering is intellectually coherent but makes the manuscript dense and occasionally hard to follow for readers primarily interested in the ontology.
• Consider restructuring so that the ontology is clearly central: a shorter context section on EUPH and PMS, then a dedicated core section on ontology design, sources, reuse strategy, domain coverage, and reasoning capabilities, followed by a separate section on implementation in EUPH and user-facing tools.
• Some current content is quite implementation heavy (e.g. detailed SOP references, UI-level workflow) and could be summarised or moved to Supplementary Material, with the main text focusing more on knowledge engineering aspects and use cases.

Thank you for your comment. We acknowledge that the manuscript describes three interconnected layers — the EUPH platform, the metadata standard, and the ontology — and that this density may obscure the ontology’s centrality. We have considered the reviewer’s suggestion to restructure the manuscript around the ontology as the core subject. The three-layer structure is, however, intrinsic to the EUPH-CV’s design: the ontology was built specifically to serve as the semantic backbone of the metadata standard and the data platform, and describing it in isolation would misrepresent its purpose and mode of deployment. Instead, we have focused our revision on sharpening the narrative within the existing structure. We integrated a first paragraph into the Discussion to underpin the rationale for the ontology design, provide worked examples of data harmonisation, and address external discoverability. We believe this approach preserves the article's intellectual coherence while making the ontology’s contribution more immediately legible to readers.

3. Practical demonstration of value for data integration
Reviewer 2 already requested a “specific example of data standardization/harmonization/integration that demonstrates the value of this ontology,” and the authors indicate that they added Pollination as a proof-of-concept dataset.
• At present, the Pollination example is only briefly mentioned as a dataset annotated with the ontology; there is no concrete, step by step illustration of how ontology terms (classes, IRIs, relations) were used to harmonise heterogeneous fields, nor what kinds of queries or interoperability become possible as a result.
• For a research article, at least one worked example is needed: show a small subset of real pollinator datasets before and after mapping to EUPH CV (e.g. different field names and vocabularies for species, hive metrics, pesticide exposures), then demonstrate how ontology based annotation enables unified querying or cross study aggregation that was not feasible before.
Without such an example, many of the claims about improved standardisation and FAIRness remain somewhat aspirational.

Thank you for your comment. This point is well taken and constitutes the most substantive revision in this round. We have added a concrete, step-by-step worked example to the Discussion illustrating how three independently collected datasets on Varroa infestation — recorded in Belgian Dutch, Arabic, and Spanish, with different field names and different units — can be harmonised through mapping to shared EUPH-CV IRIs, OWL-based class hierarchies, and UO unit annotations. The example explicitly shows what becomes possible after ontology-based annotation: a unified SPARQL query retrievable via the EUPH API, cross-language aggregation without manual intervention, and downstream comparability. The example uses realistic EUPH codes and IRIs that can be verified directly in the live ontology.

4. Ontology design choices and governance
The ontology uses BFO as an upper ontology in newer sections and imports from multiple OBO and related ontologies, while also introducing “private classes” for source specific terms (e.g. FAOSTAT, EFSA) linked via equivalentClass.
• The paper notes that domain partitioning is by scientific field and that some areas still reflect an older “lexical” structure predating full ontology design. This hybrid status is realistic but should be explicitly described as a current limitation and future work item: which parts are fully BFO compliant, which are still legacy, and how migration will proceed.
• The introduction of private classes and local equivalents (e.g. FAOSTAT’s Bees vs EUPH honey bee colony) is understandable, but the strategy and potential for duplication/confusion need clearer explanation: how will users know which class to use when annotating new data, and how will changes in external vocabularies be managed over time?
• Governance and community processes are mentioned (BeeLife, Apimondia WG, SOP 012 and SOP 016), but the criteria for accepting new classes, resolving conflicts, or deprecating terms could be more concretely illustrated.
This is important if the authors want to claim MIRO compliance and long term sustainability.

During the review process, extensive work on the Pollinator Ontology has continued. BFO has been fully implemented as the upper ontology, and there is no more lexical structure outside BFO left. The hybrid status has been resolved. The domain-based structure, however, has been maintained for reasons that are discussed in the manuscript.

The concept of private versus global classes is maintained in the Pollinator Hub's controlled vocabulary. Still, its conceptual design has been modified so that it no longer affects the ontology.

The governance of the community process has been addressed in the manuscript.

5. Discoverability, interoperability and risk of “another silo”
The authors rightly emphasise w3id registration, OBO submission, and Zenodo/GitLab hosting. They also respond to concerns about EUPH becoming another silo by arguing that FAIR and standardised data, linked out to external repositories and journals, prevent this.
• It would strengthen the argument to explicitly describe how EUPH-annotated data could be discovered and reused from outside the EUPH environment. For example, how would a phenology database or a global pollinator trait database programmatically discover relevant classes and map their own fields to EUPH CV IRIs?
• The current text still focuses mainly on what happens within EUPH; more emphasis on external machine actionable access (e.g. through OLS, BioPortal, APIs) and some concrete interoperability scenarios would make the “not a silo” claim more convincing.

We have added paragraphs to the Discussion that specifically address external discoverability and machine-actionable interoperability outside the EUPH environment. It describes concretely how an external researcher — for example, operating a phenology monitoring database — can: (1) search Pollinator Ontology terms in OBO Foundry once it has been approved by the BioPortal and other portals; (2) retrieve full class definitions, relationships and translations in JSON-LD format from the EUPH API; and (3) submit missing terms through the community contribution interface. It also explains that annotated datasets on EUPH are exposed as RDF triples, enabling programmatic harvesting by global aggregators such as GBIF. We have removed the informal phrasing. We hope this revision makes the “not a silo” claim substantively convincing rather than merely asserted.

Minor and stylistic points
• Some numerical descriptions (counts of classes, translations, relationships) are helpful but could be streamlined; the key message is coverage and balance across domains, not the exact numbers.
• There are occasional small inconsistencies (e.g. slightly different class counts in main text vs tracked changes portion) that should be checked for coherence.
• The English is generally clear, but a final editorial pass would help tighten long sentences and remove informal asides (e.g. “we do not really understand how it could be another data silo”).

Thanks for the comments. This has been reviewed. However, we think the reviewer is mixing the manuscript draft with the response to reviewers.

Suggested recommendation
On balance, this is a valuable community resource and clearly within the scope of a methods/infrastructure paper, but it still requires more focused exposition and a stronger, concrete demonstration of added value for data integration.
I would recommend major revision, with the main priorities:
• Re centring the manuscript on the ontology itself (design, scope, reuse, reasoning, governance) rather than the broader platform.
• Providing at least one clear, worked example of ontology enabled data harmonisation/integration.

This has been done

• Clarifying conceptual design choices (domain based structure, legacy vs BFO based sections, private vs global classes) and how they will be managed as the ontology evolves.

During the review process, extensive work on the Pollinator Ontology has continued. BFO has been fully implemented as the upper ontology, and no legacy structure outside BFO remains. The domain-based structure, however, has been maintained for reasons that are discussed in the manuscript. The manuscript also notes that measures have been taken to ensure that the ontology's future development is not compromised. The concept of private versus global classes is maintained in the Pollinator Hub's controlled vocabulary. Still, its conceptual design has been modified so that it no longer affects the ontology.

• Expanding on external discoverability and real world interoperability to address the “data silo” concern more convincingly.

See answer above

7. PLOS authors have the option to publish the peer review history of their article ([what does this mean?](https://track.editorialmanager.com/CL0/https:%2F%2Fjournals.plos.org%2Fplosone%2Fs%2Feditorial-and-peer-review-process%23loc-peer-review-history/1/010f019cdc0c4ad3-9cbc2002-310c-4ba3-b4f7-4b8964cb94dc-000000/R3knlLGbhcrHxAlyd0HDfBOtaLsH1JxrD6u-z8EIX3Q=253)). If published, this will include your full peer review and any attached files.


If you choose “no”, your identity will remain anonymous but your review may still be made public.


**Do you want your identity to be public for this peer review?** For information about this choice, including consent withdrawal, please see our [Privacy Policy](https://track.editorialmanager.com/CL0/https:%2F%2Fwww.plos.org%2Fprivacy-policy/1/010f019cdc0c4ad3-9cbc2002-310c-4ba3-b4f7-4b8964cb94dc-000000/g0TOL6MRzIq8g7YUgfh7iOxWO6cEFmwlAhJASq-Qbz4=253).

Reviewer #4: No

Reviewer #5: **Yes:**
