# Supplementary material for: The EU pollinator hub controlled vocabulary: An ontology for pollinators
Source: PLoS One. 2026 Aug 26;21(8):e0353382. doi: 10.1371/journal.pone.0353382 (PMC13514042; doi:10.1371/journal.pone.0353382)
Supplement: Peer review history

# Peer review: Decision Letter 0

**DOI:** 10.1371/journal.pone.0353382.r001

**Authors:** Sergio Consoli

22 Oct 2025

Dear Dr. Simon Delso,

Thank you for submitting your manuscript to PLOS ONE. After careful consideration, we feel that it has merit but does not fully meet PLOS ONE’s publication criteria as it currently stands. Therefore, we invite you to submit a revised version of the manuscript that addresses the points raised during the review process.

Please submit your revised manuscript by Dec 06 2025 11:59PM. If you will need more time than this to complete your revisions, please reply to this message or contact the journal office at plosone@plos.org. When you're ready to submit your revision, log on to https://www.editorialmanager.com/pone/ and select the 'Submissions Needing Revision' folder to locate your manuscript file.

- A rebuttal letter that responds to each point raised by the academic editor and reviewer(s). You should upload this letter as a separate file labeled 'Response to Reviewers'.
- A marked-up copy of your manuscript that highlights changes made to the original version. You should upload this as a separate file labeled 'Revised Manuscript with Track Changes'.
- An unmarked version of your revised paper without tracked changes. You should upload this as a separate file labeled 'Manuscript'.

If you would like to make changes to your financial disclosure, please include your updated statement in your cover letter. Guidelines for resubmitting your figure files are available below the reviewer comments at the end of this letter.

If applicable, we recommend that you deposit your laboratory protocols in protocols.io to enhance the reproducibility of your results. Protocols.io assigns your protocol its own identifier (DOI) so that it can be cited independently in the future. For instructions see: https://journals.plos.org/plosone/s/submission-guidelines#loc-laboratory-protocols. Additionally, PLOS ONE offers an option for publishing peer-reviewed Lab Protocol articles, which describe protocols hosted on protocols.io. Read more information on sharing protocols at https://plos.org/protocols?utm_medium=editorial-email&utm_source=authorletters&utm_campaign=protocols.

We look forward to receiving your revised manuscript.

Kind regards,

Sergio Consoli

Academic Editor

PLOS ONE

Journal Requirements:

When submitting your revision, we need you to address these additional requirements.

1.Please ensure that your manuscript meets PLOS ONE's style requirements, including those for file naming. The PLOS ONE style templates can be found at

https://journals.plos.org/plosone/s/file?id=wjVg/PLOSOne_formatting_sample_main_body.pdf and

https://journals.plos.org/plosone/s/file?id=ba62/PLOSOne_formatting_sample_title_authors_affiliations.pdf

2. We note that the grant information you provided in the ‘Funding Information’ and ‘Financial Disclosure’ sections do not match.

When you resubmit, please ensure that you provide the correct grant numbers for the awards you received for your study in the ‘Funding Information’ section.

3. Please note that funding information should not appear in any section or other areas of your manuscript. We will only publish funding information present in the Funding Statement section of the online submission form. Please remove any funding-related text from the manuscript.

4. Thank you for uploading your study's underlying data set. Unfortunately, the repository you have noted in your Data Availability statement does not qualify as an acceptable data repository according to PLOS's standards.

At this time, please upload the minimal data set necessary to replicate your study's findings to a stable, public repository (such as figshare or Dryad) and provide us with the relevant URLs, DOIs, or accession numbers that may be used to access these data. For a list of recommended repositories and additional information on PLOS standards for data deposition, please see https://journals.plos.org/plosone/s/recommended-repositories.

5. We note that your Data Availability Statement is currently as follows: “”All relevant data are within the manuscript and its Supporting Information files. The EU Pollinator Hub Controlled Vocabulary (EUPH-CV) is openly accessible at https://app.pollinatorhub.eu/vocabulary. Metadata and versioned releases of the ontology are available under a Creative Commons Attribution 4.0 license via the BeeLife repository at https://gitlab.com/bee-life/pollinator-hub.

Please confirm at this time whether or not your submission contains all raw data required to replicate the results of your study. Authors must share the “minimal data set” for their submission. PLOS defines the minimal data set to consist of the data required to replicate all study findings reported in the article, as well as related metadata and methods (https://journals.plos.org/plosone/s/data-availability#loc-minimal-data-set-definition).

For example, authors should submit the following data:

- The values behind the means, standard deviations and other measures reported;

- The values used to build graphs;

- The points extracted from images for analysis.

Authors do not need to submit their entire data set if only a portion of the data was used in the reported study.

If your submission does not contain these data, please either upload them as Supporting Information files or deposit them to a stable, public repository and provide us with the relevant URLs, DOIs, or accession numbers. For a list of recommended repositories, please see https://journals.plos.org/plosone/s/recommended-repositories.

If there are ethical or legal restrictions on sharing a de-identified data set, please explain them in detail (e.g., data contain potentially sensitive information, data are owned by a third-party organization, etc.) and who has imposed them (e.g., an ethics committee). Please also provide contact information for a data access committee, ethics committee, or other institutional body to which data requests may be sent. If data are owned by a third party, please indicate how others may request data access.

6. We notice that your supplementary figures are uploaded with the file type 'Figure'. Please amend the file type to 'Supporting Information'. Please ensure that each Supporting Information file has a legend listed in the manuscript after the references list.

7. If the reviewer comments include a recommendation to cite specific previously published works, please review and evaluate these publications to determine whether they are relevant and should be cited. There is no requirement to cite these works unless the editor has indicated otherwise.

Additional Editor Comments:

Please pay particular attention to the comments provided by Reviewer 2, as they offer important insights and suggest practical improvements. In particular there is need for enhanced documentation of the ontology’s functionality and adoption strategy, emphasizing the importance of using standards like MIRO for ontology reporting, and the necessity to demonstrate the ontology’s value through specific examples of data integration. Additionally, technical concerns such as the discoverability of the ontology and improvements to the user interface should be addressed to ensure that the tool is effective and easily adoptable by the research community. By focusing on these areas, your revised manuscript will be better aligned with the publication criteria of PLOS ONE.

[Note: HTML markup is below. Please do not edit.]

Reviewer's Responses to Questions

**Comments to the Author**

1. Is the manuscript technically sound, and do the data support the conclusions?

Reviewer #1: Yes

Reviewer #2: No

Reviewer #3: Yes

**********

2. Has the statistical analysis been performed appropriately and rigorously?

Reviewer #1: N/A

Reviewer #2: N/A

Reviewer #3: N/A

**********

3. Have the authors made all data underlying the findings in their manuscript fully available?

The PLOS Data policy

Reviewer #1: Yes

Reviewer #2: No

Reviewer #3: Yes

**********

4. Is the manuscript presented in an intelligible fashion and written in standard English?

Reviewer #1: Yes

Reviewer #2: Yes

Reviewer #3: Yes

**********

Reviewer #1: The manuscript demonstrates a thoughtful integration of empirical evidence and conceptual clarity, and with further refinement in structure and argument flow, it holds strong potential to make a meaningful contribution to the field.

Reviewer #2: In order to be accepted, this paper needs much more information about the ontology itself and how the ontology improves data standardization/integration. I would recommend looking at the MIRO (minimum information for reporting an ontology) standard. I am not convinced that an ontology is the most appropriate knowledge modeling structure to use here. Perhaps a knowledge graph would be better? Specific suggestions below.

1. Is this ontology findable via OLS, BioPortal, or OBO Foundry? It will not help standardize data if it is not adopted and to be adopted people have to know it exists.

2. I would like to see a specific example of data standardization/harmonization/integration that demonstrates the value of this ontology

3. In Fig 2, it is not clear what someone would do to initiate an edit and what kinds of edits are possible. I am also not convinced that your interface is better than using GitHub for collaborative ontology development.

4. Have you registered your namespace with w3id.org? Probably better than managing EUPH identifiers yourself.

5. In table 1, who made the decision of which classes were root and secondary? Also, are the agricultural classes reusing agricultural ontologies?

6. I would recommend adhering to the domain division rule for ontologies. Use a knowledge graph to traverse domains.

7. Where is the ontology OWL file?

8. You don't want to make EUPH another data silo. The ontology is supposed to help avoid this. How would data from EUPH integrate with data from related knowledgebases, such as a phenology-focused data resource, for example?

Reviewer #3: PollinatorHub is a necessary initiative and, at the same time, a huge project. As a reviewer, I agree with the authors of this publication that there is a great deal of diverse data on pollinating insects, but it is extremely fragmented, not standardised, and therefore of limited use to others.

My only minor suggestion to the authors would be to add an example procedure to the article, perhaps in the form of a diagram, on how to enter one's own scientific data into the PollinatorHub database. In other words, a practical guide for the scientist user. The article is written in very technical language, so an example would probably be very helpful for the readers of this article.

**********

what does this mean?). If published, this will include your full peer review and any attached files.

If you choose “no”, your identity will remain anonymous but your review may still be made public.

**Do you want your identity to be public for this peer review?**  For information about this choice, including consent withdrawal, please see our Privacy Policy

Reviewer #1: **Yes:** Charles Fernando dos Santos

Reviewer #2: **Yes:** Anne E Thessen

Reviewer #3: No

**********

[NOTE: If reviewer comments were submitted as an attachment file, they will be attached to this email and accessible via the submission site. Please log into your account, locate the manuscript record, and check for the action link "View Attachments". If this link does not appear, there are no attachment files.]

To ensure your figures meet our technical requirements, please review our figure guidelines: https://journals.plos.org/plosone/s/figures

You may also use PLOS’s free figure tool, NAAS, to help you prepare publication quality figures: https://journals.plos.org/plosone/s/figures#loc-tools-for-figure-preparation.

NAAS will assess whether your figures meet our technical requirements by comparing each figure against our figure specifications.

# Author comment: Author response to Decision Letter 1

**DOI:** 10.1371/journal.pone.0353382.r002

25 Dec 2025

REVIEW OF RUBINIGG ET AL

1. Please ensure that your manuscript meets PLOS ONE's style requirements, including those for file naming. The PLOS ONE style templates can be found at

https://journals.plos.org/plosone/s/file?id=wjVg/PLOSOne_formatting_sample_main_body.pdf and

https://journals.plos.org/plosone/s/file?id=ba62/PLOSOne_formatting_sample_title_authors_affiliations.pdf

Style modifications have been performed to meet PLOS ONE’s style requirements

2. We note that the grant information you provided in the ‘Funding Information’ and ‘Financial Disclosure’ sections do not match.

When you resubmit, please ensure that you provide the correct grant numbers for the awards you received for your study in the ‘Funding Information’ section.

This was modified, thank you

3. Please note that funding information should not appear in any section or other areas of your manuscript. We will only publish funding information present in the Funding Statement section of the online submission form. Please remove any funding-related text from the manuscript.

This was removed, thank you

4. Thank you for uploading your study's underlying data set. Unfortunately, the repository you noted in your Data Availability statement does not meet PLOS's standards for an acceptable data repository.

At this time, please upload the minimal dataset necessary to replicate your study's findings to a stable, public repository (such as figshare or Dryad) and provide us with the relevant URLs, DOIs, or accession numbers that can be used to access these data. For a list of recommended repositories and additional information on PLOS standards for data deposition, please see https://journals.plos.org/plosone/s/recommended-repositories.

The data have been included in the Zenodo repository, in addition to the EU Pollinator Hub, and the links have been included in the Manuscript.

5. We note that your Data Availability Statement is currently as follows: “ All relevant data are within the manuscript and its Supporting Information files. The EU Pollinator Hub Controlled Vocabulary (EUPH-CV) is openly accessible at https://app.pollinatorhub.eu/vocabulary. Metadata and versioned releases of the ontology are available under a Creative Commons Attribution 4.0 license via the BeeLife repository at https://gitlab.com/bee-life/pollinator-hub.

Please confirm at this time whether or not your submission contains all the raw data required to replicate the results of your study. Authors must share the “minimal data set” for their submission. PLOS defines the minimal data set as the data required to replicate all study findings reported in the article, along with related metadata and methods (https://journals.plos.org/plosone/s/data-availability#loc-minimal-data-set-definition).

For example, authors should submit the following data:

- The values behind the means, standard deviations and other measures reported;

- The values used to build graphs;

- The points extracted from images for analysis.

Authors do not need to submit their entire dataset if only a portion of it was used in the reported study.

If your submission does not contain these data, please either upload them as Supporting Information files or deposit them to a stable, public repository and provide us with the relevant URLs, DOIs, or accession numbers. For a list of recommended repositories, please see https://journals.plos.org/plosone/s/recommended-repositories.

If there are ethical or legal restrictions on sharing a de-identified data set, please explain them in detail (e.g., data contain potentially sensitive information, data are owned by a third-party organization, etc.) and who has imposed them (e.g., an ethics committee). Please also provide contact information for a data access committee, ethics committee, or other institutional body to which data requests may be sent. If data are owned by a third party, please indicate how others may request data access.

The resubmission includes all raw data and links to the raw data needed to replicate the study's results. The dataset is entirely available in the provided links, which are included in the reviewed manuscript. The Data were made available at Zotero in addition to EUPH (see references in the Manuscript).

6. We notice that your supplementary figures are uploaded with the file type 'Figure'. Please amend the file type to 'Supporting Information'. Please ensure that each Supporting Information file has a legend listed in the manuscript after the references list.

There are no supplementary figures, so this comment may refer to the figures to be included in the main manuscript. Yet, we do not understand this comment, because we do not know which figures are referred to.

7. If the reviewer comments include a recommendation to cite specific previously published works, please review and evaluate these publications to determine whether they are relevant and should be cited. There is no requirement to cite these works unless the editor has indicated otherwise.

Additional Editor Comments:

Please pay particular attention to the comments provided by Reviewer 2, as they offer important insights and suggest practical improvements. In particular, there is a need for enhanced documentation of the ontology’s functionality and adoption strategy, emphasising the importance of using standards like MIRO for ontology reporting, and the necessity to demonstrate the ontology’s value through specific examples of data integration. Additionally, technical concerns such as the discoverability of the ontology and improvements to the user interface should be addressed to ensure that the tool is effective and easily adoptable by the research community. By focusing on these areas, your revised manuscript will be better aligned with the publication criteria of PLOS ONE.

[Note: HTML markup is below. Please do not edit.]

Reviewer's Responses to Questions

Comments to the Author

1. Is the manuscript technically sound, and do the data support the conclusions?

The manuscript must describe a technically sound piece of scientific research with data that supports the conclusions. Experiments must have been conducted rigorously, with appropriate controls, replication, and sample sizes. The conclusions must be drawn appropriately based on the data presented.

Reviewer #1: Yes

Reviewer #2: No

Reviewer #3: Yes

2. Has the statistical analysis been performed appropriately and rigorously?

Reviewer #1: N/A

Reviewer #2: N/A

Reviewer #3: N/A

3. Have the authors made all data underlying the findings in their manuscript fully available?

The PLOS Data policy requires authors to make all data underlying the findings described in their manuscript fully available without restriction, with rare exception (please refer to the Data Availability Statement in the manuscript PDF file). The data should be provided as part of the manuscript or its supporting information, or deposited to a public repository. For example, in addition to summary statistics, the data points behind means, medians and variance measures should be available. If there are restrictions on publicly sharing data—e.g. participant privacy or use of data from a third party—those must be specified.

Reviewer #1: Yes

Reviewer #2: No

Reviewer #3: Yes

4. Is the manuscript presented in an intelligible fashion and written in standard English?

PLOS ONE does not copyedit accepted manuscripts, so the language in submitted articles must be clear, correct, and unambiguous. Any typographical or grammatical errors should be corrected at revision, so please note any specific errors here.

Reviewer #1: Yes

Reviewer #2: Yes

Reviewer #3: Yes

5. Review Comments to the Author

Please use the space provided to explain your answers to the questions above. You may also include additional comments for the author, including concerns about dual publication, research ethics, or publication ethics. (Please upload your review as an attachment if it exceeds 20,000 characters)

Reviewer #1: The manuscript demonstrates a thoughtful integration of empirical evidence and conceptual clarity, and with further refinement in structure and argument flow, it holds strong potential to make a meaningful contribution to the field.

Thank you!

Reviewer #2: In order to be accepted, this paper needs much more information about the ontology itself and how the ontology improves data standardization/integration. I would recommend looking at the MIRO (minimum information for reporting an ontology) standard. I am not convinced that an ontology is the most appropriate knowledge modelling structure to use here. Perhaps a knowledge graph would be better? Specific suggestions below.

The MIRO standard has been fully implemented, and its implementation has been discussed in the manuscript. At the time of manuscript submission, the ontology was still underdeveloped in some respects, particularly regarding the actual purpose of ontologies. The work of the last few months has significantly improved this, and we hope that the reviewer will appreciate these efforts.

1. Is this ontology findable via OLS, BioPortal, or OBO Foundry? It will not help standardize data if it is not adopted and to be adopted people have to know it exists.

The ontology was self-published in the EU Pollinator Hub and presented in relevant fora (Apimondia, pollinator-related projects, etc.). It has also been submitted to OBO Foundry, and importing to ontology lookup services requires the creation of an OWL file, which has also been done.

2. I would like to see a specific example of data standardization/harmonization/integration that demonstrates the value of this ontology

We have included an example in the manuscript.

3. In Fig 2, it is not clear what someone would do to initiate an edit and what kinds of edits are possible. I am also not convinced that your interface is better than using GitHub for collaborative ontology development.

We have added a section in the manuscript to justify the use of a native user interface rather than a development platform such as GitHub, and we have added a link to the EUPH product directory.

4. Have you registered your namespace with w3id.org? Probably better than managing EUPH identifiers yourself.

Thank you for this suggestion, we did it. The merge was successful, three namespaces are now registered and pointing to the Pollinator Hub:

https://w3id.org/euph links directly to repository,

https://w3id.org/euph/{id} links relative to class id, for example, https://w3id.org/euph/3344 links to the class with code 3344)

https://w3id.org/pms/{name} links relative to a descriptor with given name that uses the ontology. For example https://w3id.org/pms/hiveWeight will open a class with the name hiveWeight (currently not implemented on our side)

5. In Table 1, who made the decision of which classes were root and secondary? Also, are the agricultural classes reusing agricultural ontologies?

The construction of root trees as well as the assignment of classes to a domain are regulated in SOP 012, section 5.2.2.5 (Ontology design). An explanation has been added in the manuscript. Regarding agricultural ontologies, some agricultural domains have indeed been integrated into the EUPH-CV. A short note has been added to the manuscript. Unfortunately, ontologies in this domain are often poorly developed and incomplete, especially in beekeeping, which makes it difficult to reuse them to a greater extent and was one of the reasons for building this ontology, as stated in the manuscript.

6. I would recommend adhering to the domain division rule for ontologies. Use a knowledge graph to traverse domains.

Thank you for the suggestion. The knowledge graph to traverse domains will be included in the OWL file

7. Where is the ontology OWL file?

The reviewer refers to this source: https://www.w3.org/TR/owl2-syntax/

8. You don't want to make EUPH another data silo. The ontology is supposed to help avoid this. How would data from EUPH integrate with data from related knowledge bases, such as a phenology-focused data resource, for example?

The EUPH aims to create a data repository that enables validation of the FAIRness and Quality of the data uploaded there, and then pushes for the standardisation of the data uploaded to it (which is why the PMS was created and integrated with other internationally available standards such as Darwin Core, ISO, etc.). The data is linked to Zenodo (supported by CERN and ensuring long-term preservation), and we are working to link it to several Data Journals, so users can choose to have their data in multiple repositories. This increases data redundancy and resilience. A data silo is created when the available data is not FAIR and cannot be interoperated with or reused. In essence, the very creation of the EUPH is to avoid this, so we do not really understand how it could be another data silo. We have to better explain this in the discussion.

Reviewer #3: PollinatorHub is a necessary initiative and, at the same time, a huge project. As a reviewer, I agree with the authors of this publication that there is a great deal of diverse data on pollinating insects, but it is extremely fragmented, not standardised, and therefore of limited use to others.

My only minor suggestion to the authors would be to add an example procedure to the article, perhaps in the form of a diagram, on how to enter one's own scientific data into the PollinatorHub database. In other words, a practical guide for the scientist user. The article is written in very technical language, so an example would probably be very helpful for the readers of this article.

Thank you for the comments. This comment does not refer to the ontology but to the data upload. A guide is available while users are uploading data. Some SOPs refer to the data upload process (namely, 6, 9, 8, 12, and 13), and video tutorials show the procedure (https://www.youtube.com/watch?v=YNTI_capc1k&list=PLGe_DfwrNqSJkSPyUfyKseGof71h1oD5f&index=4). We are also working on a user-friendly user guide showing the step-by-step process.

Indeed, it may not be evident to the users. We have improved its accessibility so that potential users find it easier to upload their data.

6. PLOS authors have the option to publish the peer review history of their article (what does this mean?). If published, this will include your full peer review and any attached files.

If you choose “no”, your identity will remain anonymous but your review may still be made public.

Do you want your identity to be public for this peer review? For information about this choice, including consent withdrawal, please see our Privacy Policy.

Reviewer #1: Yes: Charles Fernando dos Santos

Reviewer #2: Yes: Anne E Thessen

Reviewer #3: No

Attachment Submitted filename: Rubinigg et al. - Response to Reviewers.pdf

# Peer review: Decision Letter 1

**DOI:** 10.1371/journal.pone.0353382.r003

**Authors:** Samuel Babarinde

11 Mar 2026

Thank you for submitting your manuscript to PLOS ONE. After careful consideration, we feel that it has merit but does not fully meet PLOS ONE’s publication criteria as it currently stands. Therefore, we invite you to submit a revised version of the manuscript that addresses the points raised during the review process.

Please submit your revised manuscript by Apr 25 2026 11:59PM. If you will need more time than this to complete your revisions, please reply to this message or contact the journal office at plosone@plos.org. When you're ready to submit your revision, log on to https://www.editorialmanager.com/pone/ and select the 'Submissions Needing Revision' folder to locate your manuscript file.

- A letter that responds to each point raised by the academic editor and reviewer(s). You should upload this letter as a separate file labeled 'Response to Reviewers'.
- A marked-up copy of your manuscript that highlights changes made to the original version. You should upload this as a separate file labeled 'Revised Manuscript with Track Changes'.
- An unmarked version of your revised paper without tracked changes. You should upload this as a separate file labeled 'Manuscript'.

If you would like to make changes to your financial disclosure, please include your updated statement in your cover letter. Guidelines for resubmitting your figure files are available below the reviewer comments at the end of this letter.

If applicable, we recommend that you deposit your laboratory protocols in protocols.io to enhance the reproducibility of your results. Protocols.io assigns your protocol its own identifier (DOI) so that it can be cited independently in the future. For instructions see: https://journals.plos.org/plosone/s/submission-guidelines#loc-laboratory-protocols. Additionally, PLOS ONE offers an option for publishing peer-reviewed Lab Protocol articles, which describe protocols hosted on protocols.io. Read more information on sharing protocols at https://plos.org/protocols?utm_medium=editorial-email&utm_source=authorletters&utm_campaign=protocols.

We look forward to receiving your revised manuscript.

Kind regards,

Samuel Adelani Babarinde, PhD

Academic Editor

PLOS One

Journal Requirements:

If the reviewer comments include a recommendation to cite specific previously published works, please review and evaluate these publications to determine whether they are relevant and should be cited. There is no requirement to cite these works unless the editor has indicated otherwise.

[Note: HTML markup is below. Please do not edit.]

Reviewers' comments:

Reviewer's Responses to Questions

**Comments to the Author**

Reviewer #4: All comments have been addressed

Reviewer #5: All comments have been addressed

**********

2. Is the manuscript technically sound, and do the data support the conclusions?

Reviewer #4: Yes

Reviewer #5: Partly

**********

3. Has the statistical analysis been performed appropriately and rigorously?

Reviewer #4: N/A

Reviewer #5: Yes

**********

4. Have the authors made all data underlying the findings in their manuscript fully available?

The PLOS Data policy

Reviewer #4: Yes

Reviewer #5: Yes

**********

5. Is the manuscript presented in an intelligible fashion and written in standard English?

Reviewer #4: Yes

Reviewer #5: No

**********

Reviewer #4: I think the manuscript "The EU Pollinator Hub Controlled Vocabulary: An Ontology for Pollinators" is a valuable addition to achieve usability of the much-needed Pollinator hub. I find that previous comments are answered and the manuscript is ready for consideration of accepting for publishing.

Reviewer #5: The manuscript presents a substantial and timely effort to develop a pollinator-focused ontology and controlled vocabulary, but it still needs clearer articulation of its novelty, stronger demonstration of practical utility, and better separation between platform description and ontology reporting to fully meet article-level standards.

Overall assessment

• The work is important: an openly available ontology for pollinators and beekeeping, tightly linked to a metadata standard (PMS) and implemented within the EU Pollinator Hub, directly addresses well-known problems of fragmented, non-standardised pollinator data.

• The resource is already reasonably mature (thousands of classes, 80+ languages, reuse of multiple OBO and related ontologies, versioning and SOPs), and the authors now align the description with MIRO and FAIR principles, which is appropriate for PLOS ONE.

• However, the manuscript still reads partly as a technical project report for the whole EUPH ecosystem, with relatively limited, concrete evidence of how the ontology itself improves data integration in practice, and with some conceptual and structural choices that warrant clearer justification.

Major points for critique

1. Novelty and positioning of the ontology

The paper claims that this is the first ontology to explicitly cover pollinators and beekeeping at this scale, and contrasts it mainly with the PIP vocabulary. While that is plausible, the argument is underdeveloped.

• The manuscript should more explicitly position EUPH CV and the Pollinator Ontology relative to existing biodiversity, agricultural and environmental ontologies (e.g. ENVO, AEO, crop/pollination vocabularies), beyond a list of reused sources.

• The cross domain scope (ecology, law, economics, data standards, etc.) is presented as a strength, but it risks making the ontology conceptually diffuse. The justification for structuring primarily by “scientific domain” rather than by upper ontology categories is brief and could be expanded to show why this is not just expedient but conceptually sound for pollinator data.

In its current form, the manuscript somewhat underplays the conceptual design decisions and overemphasises descriptive metrics (counts of classes, translations, etc.).

2. Focus and structure of the article

The paper mixes three layers:

• The EU Pollinator Hub platform (data archive, SOPs, upload workflows).

• The EUPH Metadata Standard (PMS + DwC + ISO, etc.).

• The Pollinator Ontology (subset of EUPH CV).

This layering is intellectually coherent but makes the manuscript dense and occasionally hard to follow for readers primarily interested in the ontology.

• Consider restructuring so that the ontology is clearly central: a shorter context section on EUPH and PMS, then a dedicated core section on ontology design, sources, reuse strategy, domain coverage, and reasoning capabilities, followed by a separate section on implementation in EUPH and user facing tools.

• Some current content is quite implementation heavy (e.g. detailed SOP references, UI-level workflow) and could be summarised or moved to Supplementary Material, with the main text focusing more on knowledge engineering aspects and use cases.

3. Practical demonstration of value for data integration

Reviewer 2 already requested a “specific example of data standardization/harmonization/integration that demonstrates the value of this ontology,” and the authors indicate that they added Pollination as a proof-of-concept dataset.

• At present, the Pollination example is only briefly mentioned as a dataset annotated with the ontology; there is no concrete, step by step illustration of how ontology terms (classes, IRIs, relations) were used to harmonise heterogeneous fields, nor what kinds of queries or interoperability become possible as a result.

• For a research article, at least one worked example is needed: show a small subset of real pollinator datasets before and after mapping to EUPH CV (e.g. different field names and vocabularies for species, hive metrics, pesticide exposures), then demonstrate how ontology based annotation enables unified querying or cross study aggregation that was not feasible before.

Without such an example, many of the claims about improved standardisation and FAIRness remain somewhat aspirational.

4. Ontology design choices and governance

The ontology uses BFO as an upper ontology in newer sections and imports from multiple OBO and related ontologies, while also introducing “private classes” for source specific terms (e.g. FAOSTAT, EFSA) linked via equivalentClass.

• The paper notes that domain partitioning is by scientific field and that some areas still reflect an older “lexical” structure predating full ontology design. This hybrid status is realistic but should be explicitly described as a current limitation and future work item: which parts are fully BFO compliant, which are still legacy, and how migration will proceed.

• The introduction of private classes and local equivalents (e.g. FAOSTAT’s Bees vs EUPH honey bee colony) is understandable, but the strategy and potential for duplication/confusion need clearer explanation: how will users know which class to use when annotating new data, and how will changes in external vocabularies be managed over time?

• Governance and community processes are mentioned (BeeLife, Apimondia WG, SOP 012 and SOP 016), but the criteria for accepting new classes, resolving conflicts, or deprecating terms could be more concretely illustrated.

This is important if the authors want to claim MIRO compliance and long term sustainability.

5. Discoverability, interoperability and risk of “another silo”

The authors rightly emphasise w3id registration, OBO submission, and Zenodo/GitLab hosting. They also respond to concerns about EUPH becoming another silo by arguing that FAIR and standardised data, linked out to external repositories and journals, prevent this.

• It would strengthen the argument to explicitly describe how EUPH annotated data could be discovered and reused from outside the EUPH environment. For example, how would a phenology database or a global pollinator trait database programmatically discover relevant classes and map their own fields to EUPH CV IRIs?

• The current text still focuses mainly on what happens within EUPH; more emphasis on external machine actionable access (e.g. through OLS, BioPortal, APIs) and some concrete interoperability scenarios would make the “not a silo” claim more convincing.

Minor and stylistic points

• Some numerical descriptions (counts of classes, translations, relationships) are helpful but could be streamlined; the key message is coverage and balance across domains, not the exact numbers.

• There are occasional small inconsistencies (e.g. slightly different class counts in main text vs tracked changes portion) that should be checked for coherence.

• The English is generally clear, but a final editorial pass would help tighten long sentences and remove informal asides (e.g. “we do not really understand how it could be another data silo”).

Suggested recommendation

On balance, this is a valuable community resource and clearly within the scope of a methods/infrastructure paper, but it still requires more focused exposition and a stronger, concrete demonstration of added value for data integration.

I would recommend major revision, with the main priorities:

• Re centring the manuscript on the ontology itself (design, scope, reuse, reasoning, governance) rather than the broader platform.

• Providing at least one clear, worked example of ontology enabled data harmonisation/integration.

• Clarifying conceptual design choices (domain based structure, legacy vs BFO based sections, private vs global classes) and how they will be managed as the ontology evolves.

• Expanding on external discoverability and real world interoperability to address the “data silo” concern more convincingly.

**********

what does this mean?). If published, this will include your full peer review and any attached files.

If you choose “no”, your identity will remain anonymous but your review may still be made public.

**Do you want your identity to be public for this peer review?**  For information about this choice, including consent withdrawal, please see our Privacy Policy

Reviewer #4: No

Reviewer #5: **Yes:**

**********

[NOTE: If reviewer comments were submitted as an attachment file, they will be attached to this email and accessible via the submission site. Please log into your account, locate the manuscript record, and check for the action link "View Attachments". If this link does not appear, there are no attachment files.]

To ensure your figures meet our technical requirements, please review our figure guidelines: https://journals.plos.org/plosone/s/figures

You may also use PLOS’s free figure tool, NAAS, to help you prepare publication quality figures: https://journals.plos.org/plosone/s/figures#loc-tools-for-figure-preparation.

NAAS will assess whether your figures meet our technical requirements by comparing each figure against our figure specifications.

Attachment Submitted filename: Review-Recommendations-PONE-D-25-42575_R1.docx

# Author comment: Author response to Decision Letter 2

**DOI:** 10.1371/journal.pone.0353382.r004

26 Apr 2026

We would like to thank the reviewers for their feedback. We hope to have addressed all the comments in this revision, although some requested the opposite of what previous reviewers did (e.g., the level of procedural description).

We included an extensive section in the Discussion that provides context for our development, giving the manuscript a broader perspective on the ontology's impact and utility. We agree that the positioning relative to broader biodiversity and agricultural ontologies was underdeveloped, and we have substantially expanded the Discussion to address this. A new paragraph has been added that explicitly contrasts the EUPH-CV with ENVO, Darwin Core, HAO/Uberon, and crop-specific vocabularies, and articulates the three complementary dimensions that distinguish the EUPH-CV: (1) its comprehensive, domain-spanning pollinator-specific scope; (2) its multilingual layer across 88 languages; and (3) its formalisation of the beekeeping controlled vocabulary, previously existing only in specialist print dictionaries. We also clarify that the cross-domain scope is not conceptually diffuse, but rather a necessary consequence of the ecological and regulatory position of pollinators: unlike narrowly defined biological taxa, pollinators are simultaneously objects of ecological research, veterinary management, agricultural regulation, economic valuation and international policy. An ontology that does not span these domains cannot support the data integration use cases for which it was designed. Tracked changes are visible in the revised manuscript. We hope to have addressed the reviewer's comments.

We acknowledge that the manuscript describes three interconnected layers — the EUPH platform, the metadata standard, and the ontology — and that this density may obscure the ontology’s centrality. We have considered the reviewer’s suggestion to restructure the manuscript around the ontology as the core subject. The three-layer structure is, however, intrinsic to the EUPH-CV’s design: the ontology was built specifically to serve as the semantic backbone of the metadata standard and the data platform, and describing it in isolation would misrepresent its purpose and mode of deployment. Instead, we have focused our revision on sharpening the narrative within the existing structure. We integrated a first paragraph into the Discussion to underpin the rationale for the ontology design, provide worked examples of data harmonisation, and address external discoverability. We believe this approach preserves the article's intellectual coherence while making the ontology’s contribution more immediately legible to readers.

This point is well taken and constitutes the most substantive revision in this round. We have added a concrete, step-by-step worked example to the Discussion illustrating how three independently collected datasets on Varroa infestation — recorded in Belgian Dutch, Arabic, and Spanish, with different field names and different units — can be harmonised through mapping to shared EUPH-CV IRIs, OWL-based class hierarchies, and UO unit annotations. The example explicitly shows what becomes possible after ontology-based annotation: a unified SPARQL query retrievable via the EUPH API, cross-language aggregation without manual intervention, and downstream comparability. The example uses realistic EUPH codes and IRIs that can be verified directly in the live ontology.

During the review process, extensive work on the Pollinator Ontology has continued. BFO has been fully implemented as the upper ontology, and there is no more lexical structure outside BFO left. The hybrid status has been resolved. The domain-based structure, however, has been maintained for reasons that are discussed in the manuscript.

The concept of private versus global classes is maintained in the Pollinator Hub's controlled vocabulary. Still, its conceptual design has been modified so that it no longer affects the ontology.

The governance of the community process has been addressed in the manuscript.

The Discussion also addresses external discoverability and machine-actionable interoperability outside the EUPH environment. It describes concretely how an external researcher — for example, operating a phenology monitoring database — can: (1) search Pollinator Ontology terms in OBO Foundry once it has been approved by the BioPortal and other portals; (2) retrieve full class definitions, relationships and translations in JSON-LD format from the EUPH API; and (3) submit missing terms through the community contribution interface. It also explains that annotated datasets on EUPH are exposed as RDF triples, enabling programmatic harvesting by global aggregators such as GBIF. We have removed the informal phrasing. We hope this revision makes the “not a silo” claim substantively convincing rather than merely asserted.

During the review process, extensive work on the Pollinator Ontology has continued. BFO has been fully implemented as the upper ontology, and no legacy structure outside BFO remains. The domain-based structure, however, has been maintained for reasons that are discussed in the manuscript. The manuscript also notes that measures have been taken to ensure that the ontology's future development is not compromised. The concept of private versus global classes is maintained in the Pollinator Hub's controlled vocabulary. Still, its conceptual design has been modified so that it no longer affects the ontology.

We hope this revision brings the manuscript closer to publication.

Attachment Submitted filename: Response to Reviewers 2 (1).docx

# Editor report: Decision Letter 2

**DOI:** 10.1371/journal.pone.0353382.r005

**Authors:** Samuel Babarinde

23 Jun 2026

The EU Pollinator Hub Controlled Vocabulary: An Ontology for Pollinators

PONE-D-25-42575R2

Dear Dr. Delso,

We’re pleased to inform you that your manuscript has been judged scientifically suitable for publication and will be formally accepted for publication once it meets all outstanding technical requirements.

Within one week, you’ll receive an e-mail detailing the required amendments. When these have been addressed, you’ll receive a formal acceptance letter and your manuscript will be scheduled for publication.

An invoice will be generated when your article is formally accepted. Please note, if your institution has a publishing partnership with PLOS and your article meets the relevant criteria, all or part of your publication costs will be covered. Please make sure your user information is up-to-date by logging into Editorial Manager at Editorial Manager® and clicking the ‘Update My Information' link at the top of the page. For questions related to billing, please contact billing support.

If your institution or institutions have a press office, please notify them about your upcoming paper to help maximize its impact. If they’ll be preparing press materials, please inform our press team as soon as possible -- no later than 48 hours after receiving the formal acceptance. Your manuscript will remain under strict press embargo until 2 pm Eastern Time on the date of publication. For more information, please contact onepress@plos.org.

Kind regards,

Samuel Adelani Babarinde, PhD

Academic Editor

PLOS One

Additional Editor Comments (optional):

Reviewers' comments:

# Editor report: Acceptance letter

**DOI:** 10.1371/journal.pone.0353382.r006

**Authors:** Samuel Babarinde

PONE-D-25-42575R2

PLOS One

Dear Dr. Simon Delso,

I'm pleased to inform you that your manuscript has been deemed suitable for publication in PLOS One. Congratulations! Your manuscript is now being handed over to our production team.

At this stage, our production department will prepare your paper for publication. This includes ensuring the following:

* All references, tables, and figures are properly cited

* All relevant supporting information is included in the manuscript submission,

* There are no issues that prevent the paper from being properly typeset

You will receive further instructions from the production team, including instructions on how to review your proof when it is ready. Please keep in mind that we are working through a large volume of accepted articles, so please give us a few days to review your paper and let you know the next and final steps.

Lastly, if your institution or institutions have a press office, please let them know about your upcoming paper now to help maximize its impact. If they'll be preparing press materials, please inform our press team within the next 48 hours. Your manuscript will remain under strict press embargo until 2 pm Eastern Time on the date of publication. For more information, please contact onepress@plos.org.

You will receive an invoice from PLOS for your publication fee after your manuscript has reached the completed accept phase. If you receive an email requesting payment before acceptance or for any other service, this may be a phishing scheme. Learn how to identify phishing emails and protect your accounts at https://explore.plos.org/phishing.

If we can help with anything else, please email us at customercare@plos.org.

Thank you for submitting your work to PLOS ONE and supporting open access.

Kind regards,

PLOS ONE Editorial Office Staff

on behalf of

Dr. Samuel Adelani Babarinde

Academic Editor

PLOS One
